# A self-supervised learning framework for discovering cortical folding patterns under genetic influence: Application to the Anterior Cingulate Cortex

**DOI:** 10.1162/IMAG.a.987

**Published:** 2025-11-05

**Authors:** Antoine Dufournet, Julien Laval, Denis Rivière, Héloïse de Vareilles, Graham K. Murray, Arnaud Cachia, Joël Chavas, Vincent Frouin, Jean-François Mangin

**Affiliations:** Université Paris-Saclay, CEA, CNRS, NeuroSpin, Baobab, Saclay, France; Department of Psychiatry, University of Cambridge, Cambridge, United Kingdom; Université Paris Cité, LaPsyDÉ, CNRS, Paris, France; Université Paris Cité, Institut de Psychiatrie et Neurosciences de Paris (IPNP), INSERM, Paris, France

**Keywords:** brain MRI, deep learning, cortical folding pattern, multivariate GWAS, genetics

## Abstract

Cortical folding patterns can serve as a macroscopic probe for hidden events that occur during the development of the human brain. However, the large inter-individual variability of these patterns makes them very difficult to associate with developmental pathologies. We propose a framework for discovering candidate patterns under genetic influence and illustrate its use in the anterior cingulate cortex region, where the paracingulate sulcus shapes have aroused interest in psychiatry. This framework is based on a comprehensive regional representation of fold variability inferred from a self-supervised deep learning algorithm applied to 36,000 white British ancestry subjects from the UK Biobank database. This representation can be used to train linear classification models, in order to learn to discern folding patterns from labeled databases and generalize to larger databases. In our case, the generalization of paracingulate sulcus labeling on UK BioBank subjects did not lead to clear genetic associations. Secondly, we show that new loci associated with cortical folding patterns can be discovered directly from the representation. We find in the discovery cohort 4 loci for the right hemisphere and 10 loci for the left hemisphere related to specific folding patterns of the anterior cingulate cortex ($p<5\times 10^{-8}$ ). Even if only one locus is replicated on a smaller cohort of non white British ancestry subjects, many of the discovered loci have already been associated with brain anatomy or psychiatry.

## 1 Introduction

Folding of the cerebral cortex is not the result of constraints induced by the cranium during brain growth but the result of phenomena driven by the development of the cortical architecture during neurogenesis. Cortical folds are most likely generated by tensions induced by differential growth between different brain areas (Llinares-Benadero & Borrell, 2019; Tallinen et al., 2016). This differential growth is associated with a “protomap” of primary cortical folding in the periventricular zone where neural progenitor cells give rise to neurons and glial cells. The proliferation, migration, and differentiation of these progenitor cells, under the influence of genetic regulation, explain the differential growth that gives birth to sulci and gyri. Besides the differential growth, the mechanical properties of the tissue can influence its capacity to fold more or less (Llinares-Benadero & Borrell, 2019). Hence, the cortical folding patterns provide a macroscopic indicator for hidden events of genetic origin that occur during the development of the human brain (Cachia et al., 2021).

Cortical folding abnormalities have been associated with neurodevelopmental disorders, learning difficulties, and cognitive deficits (Barkovich et al., 2012; Subramanian et al., 2020). Lissencephaly or polymicrogyria are clear signatures of severe malformations with major deleterious consequences. Another kind of unusual folding patterns is also sometimes observed, however, endowed with a geometry similar to that of frequent patterns (Régis et al., 2011). These subtle variations are potentially indicative of developmental abnormalities, but research programs aimed at characterizing them are hampered by the wide variability of normal cortical folding in the general population (Mangin et al., 2016).

**Fig. 1.**
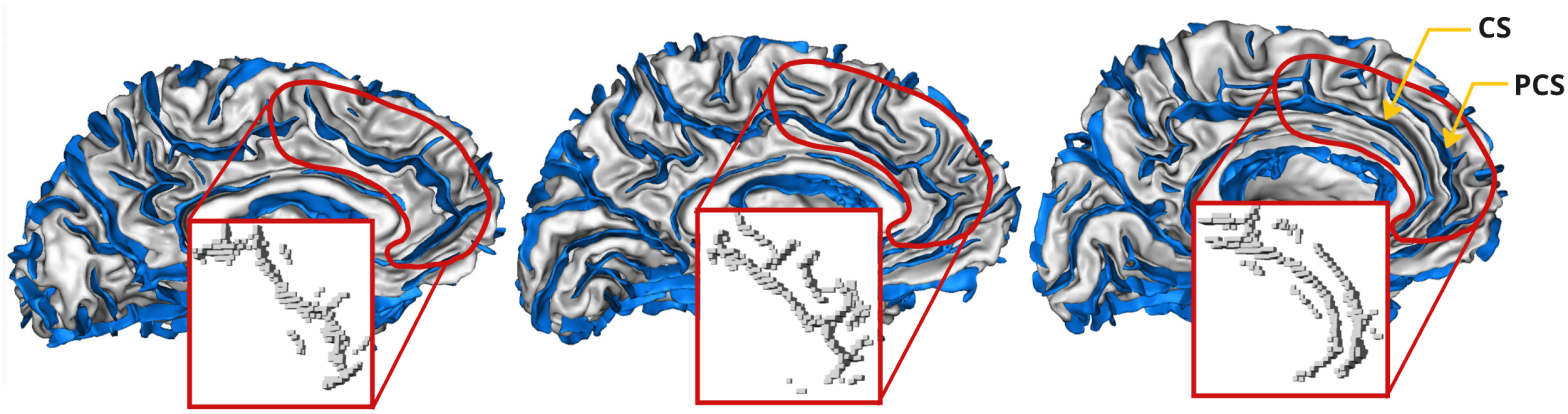
Example of diversity in the ACC for three UKB subjects. Without PCS on the left, an ambiguous case in the middle, and with PCS on the right. The sulci (in blue) are displayed together with the white matter mesh (in grey). In addition, the left ACC region obtained when masking the sulcal skeleton is delimited by the red frame.

Recent studies suggest that specific folding patterns may be associated with psychiatric disorders (Cachia et al., 2021). The Anterior Cingulate Cortex (ACC) is a key center for cognitive control and emotion regulation, making it a compelling target in this context (Tissier et al., 2018). The ACC folding patterns comprise different configurations of two key folding landmarks: the cingulate sulcus (CS) and the paracingulate sulcus (PCS). The CS always runs along the corpus callosum, while the PCS, which is not always present, is parallel to the anterior and dorsal sections of the CS (Lahutsina et al., 2022) (see Fig. 1). It has been shown that the ACC folding pattern is established prenatally and remains stable throughout life (Cachia et al., 2016). Psychotic disorders like schizophrenia or bipolar disorder are often associated with a shorter left PCS, particularly in patients with hallucinations, but the reported deviations are not consensual (Bersani et al., 2014; Fornito et al., 2006, 2008, 2009; Garrison et al., 2015, 2019; Lahutsina et al., 2022; Le Provost et al., 2003; Rametti et al., 2010; S.-C. J. Wu et al., 2024; Yücel et al., 2002).

However, the notion of PCS has limitations. By definition, it corresponds to a sulcus that runs parallel to the CS and is of sufficient length to be eligible. This simple definition leads to many ambiguous situations: indeed, the location and length of this PCS varies considerably between individuals. In subjects without a clear PCS, there is usually an alignment of small folds that are not connected but probably result from the same folding process. These correspond to the notion of sulcal roots or sulcal pits, elementary folds that constitute precursor sets to folding (Mangin et al., 2019). It seems, then, that the so-called “double parallel” configuration that corresponds to the existence of a PCS is distinguished by the initial orientation of these sulcal roots. This orientation allows the folding process to continue until several of them are connected. The CS is also often interrupted, and its subdivisions sometimes connect with the folds of the PCS potential localization, adding to the confusion.

One of the motivations of our work is to free ourselves from the complexity of the PCS, while focusing on the anatomy of the anterior cingulate cortex region, which is of interest to psychiatric research. The great diversity of possible connections between the elementary folds of this region gives rise to a large number of configurations of the ACC folding pattern (Leonard et al., 2009). Reducing this combinatorial explosion of shapes to the existence or absence of the PCS is probably too simplistic to exploit the full potential of the ACC’s folding variability. Thus, we question the optimality of this notion to describe the folding patterns of the ACC likely to indicate an increased risk of psychiatric pathology.

To provide a more exhaustive and objective description of folding patterns, we used a Self-Supervised Learning (SSL) framework called Champollion V0 (Laval et al., 2024), the architecture of which is a convolutional neural network (CNN). Based on data from 36,000 UK Biobank (UKB) subjects, it models cortical folding variability in the ACC region. SSL is ideal for describing latent variability from an objective perspective. In this article, we evaluated the folding patterns in relation to genetics, in line with (Dufournet et al., 2025). Recent studies have also explored the use of CNNs to capture the complex information embedded in MRI scans for statistical genetic analyses. For instance, Yu et al. (2024) propose an SNP-by-SNP approach where a CNN is trained to predict genotype directly from brain images, introducing a non-linear framework that challenges the traditional linear assumptions of Genome-Wide Association Study (GWAS). In our work, to better account for the complexity and high dimensionality of the phenotype, we adopt a multivariate Genome-Wide Association Study (mvGWAS) strategy.

We will use the representation provided by our SSL framework in two ways. First, with the so-called ACC dataset, manually annotated for the presence of the PCS, we will train a robust classifier to separate the PCS from non-PCS subjects. This classifier is then used to predict the PCS status of the UK BioBank subjects. Hence, thanks to the large size of the UK Biobank dataset, we perform a GWAS to assess whether the human-based annotations provided as input can be explained by genetics. In a second approach, the samples in the representation space will undergo an mvGWAS to test the extent to which the provided representation can be explained by genetics, without any kind of annotation. For each genetics hit, we will characterize the associated folding pattern variation.

## 2 Methods

### 2.1 Datasets

The data used in this paper were obtained from the UK BioBank (application number #64984). This study uses imaging and genotyping data from 42,768 UK Biobank participants (47% male), 44 to 82 years old (UK Biobank field: 53, first imaging visit), with a mean age of 64.0±7.7 years. We also used 341 subjects from the ACC dataset (54% male, 9 to 40 years old, with a mean age of 18.0±8.5 ), with manual annotation for CS and PCS (Cachia et al., 2016; Cachia et al., 2014; Chakravarty et al., 2015; Delalande et al., 2020; Rapoport & Gogtay, 2011; Tissier et al., 2018) performed with the BrainVISA software package (Rivière et al., 2022).

### 2.2 Preprocessing and folds semantic segmentation

**Fig. 2.**
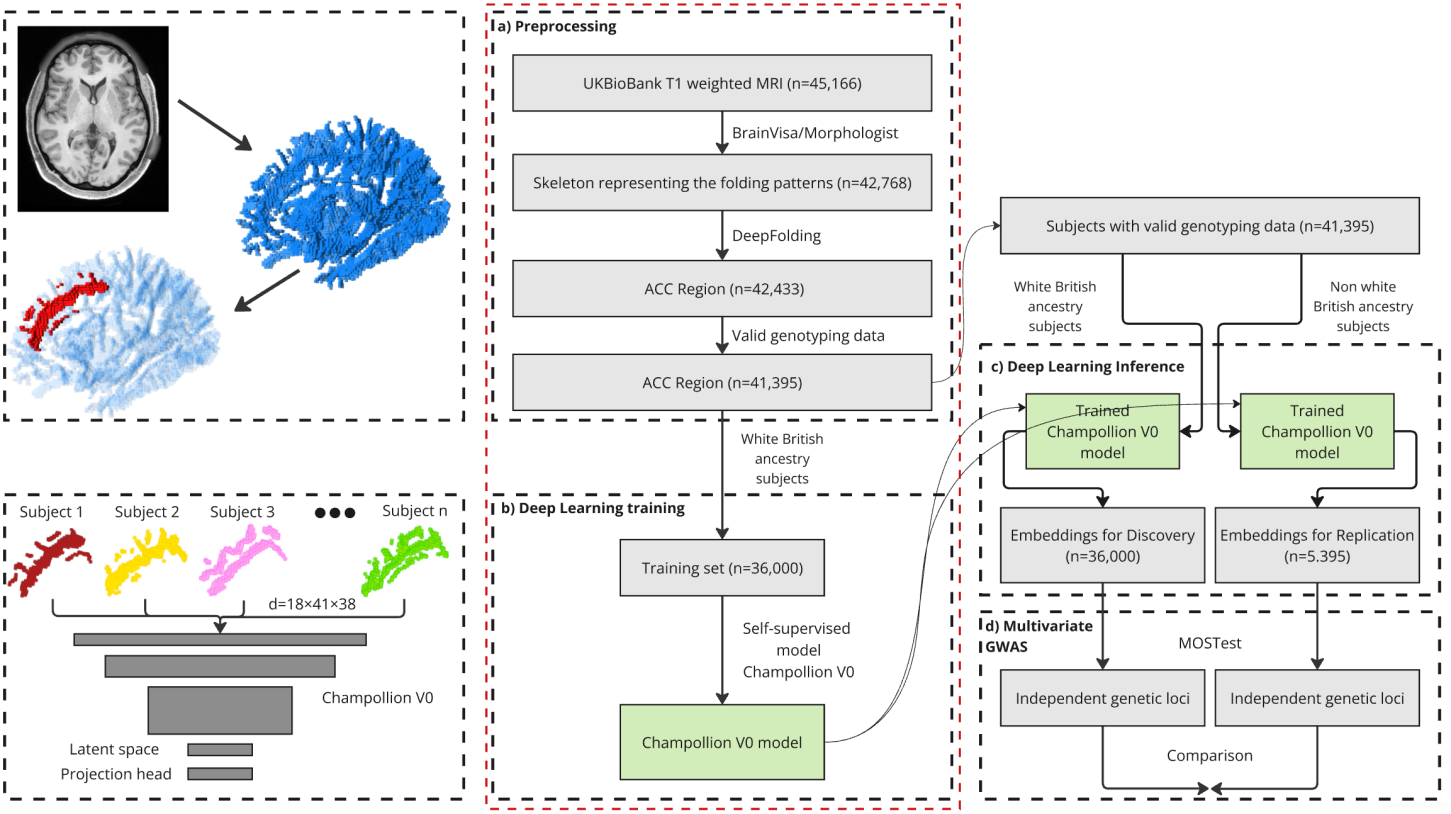
Overview of the self-supervised method, from the embeddings to the genetic association. (a) The sulcal skeleton (in blue) is extracted from the T1 image, with the sulcal region of interest (in red) highlighting the anterior cingulate cortex in this example. (b) The self-supervised model is trained exclusively on 36,000 subjects of the white British ancestry dataset using a Barlow Twin loss function. (c) The UKB subjects are encoded in the representation space using the same Champollion V0 model. (d) The MOSTest is conducted on both the 36,000-subject discovery set and the 5,395-subject replication set for comparison.

From the 42,768 T1-weighted structural images available (UK Biobank field: 20252 first imaging visit), with a resolution of 1 mm isotropic, we run the Morphologist pipeline of the BrainVISA software package^†^ (Mangin et al., 2004). This pipeline provides a 1 mm isotropic sulcal skeleton in the native space, representing a negative cast of the cortex (Mangin et al., 1995). This skeleton describes only the morphology of the cortex, in the form of a volumetric binary image. Insofar as it describes neither variations in cortical thickness nor variations in the opening of cortical folds, this description of morphology enables learning to focus on variations in cortical folding patterns. This sulcal skeleton is then affinely normalized to the MNI space. Finally, for training efficiency, we downsampled the skeleton to a 2 mm isotropic resolution sufficient to describe folding patterns. Laval et al. (2024) previously demonstrated that a 1.5 mm resolution did not yield better performance than the 2 mm resolution used in this paper. Pattern variability is illustrated in Figure 1 and the preprocessing in Figure 2a.

Then, the skeleton is masked to focus the analysis on a Region of Interest corresponding in this paper to the ACC, namely the region of the MNI reference space that encompasses the PCS of the ACC dataset and the anterior CS of BrainVISA’s nomenclature. This ACC mask was generated using the Deepfolding library and applied to subjects with manually annotated PCS and CS (Chavas et al., 2022).^‡^ The mask results from the space containing the summation of these subjects’ PCS and anterior CS skeletons, followed by a 5 mm spatial dilation. Thus, the ACC mask is expected to include the anterior CS and potential PCS of any other subject affinely normalized to the MNI space.

Subjects with a poor Morphologist segmentation (BrainVISA software package) were removed during quality control (QC). In addition, subjects with too many voxels in the sulcal skeleton (more than 1.2 times the number of voxels in the last decile) were also removed. Examples of discarded subjects are presented in Supplementary Figure S1. After QC, 42,433 subjects could be masked for the left and right ACC. Figure 1 illustrates three examples of the ACC masking process. The red outlines highlight the extracted sulcal regions, allowing a visual comparison of cortical folding variations across subjects.

### 2.3 Discovery and replication cohorts for genetic association

From UK BioBank’s imputed SNPs (Version 3) by Bycroft et al. (2018), only bi-allelic SNPs with a Minor Allele Frequency (MAF) >0.05 are considered, resulting in 6,031,842 SNPs.

Using PLINK 2.0 (Chang et al., 2015), subjects were filtered based on their ancestry with the –keep command. SNPs and individuals with missing genotype data were removed using the –geno and –mind commands with default parameters, respectively. SNPs failing the Hardy-Weinberg equilibrium test at a significance threshold of $p<1\times 10^{-15}$ (–hwe 1e-15) were excluded. Duplicated SNPs had already been removed prior to these steps.

From the 42,433 subjects preprocessed, only 41,395 subjects have valid genotyping data. In this study, the discovery cohort consisted of 36,000 participants of white British ancestry. The replication cohort comprised 5,395 participants of non-white British ancestry (UK BioBank field: 22006).

### 2.4 Champollion V0: a self-supervised deep learning model

As pointed out in the example of Figure 1, sulci shapes vary widely between subjects, not only in terms of length, depth, opening, but also in terms of relative position, intersections, interruptions, and curvatures. This is why the usual atlases of cortical folding do not reflect the full inter-individual variability. For example, morphometric measurements derived from the nomenclature used by BrainVISA software (hull junction length, surface, and mean depth of the sulci) provide only a very partial description of the variability of cortical folding in the ACC, in addition to depending on the quality of the segmentation. The framework we propose revisits the problem by leveraging the potential of recent self-supervised learning methods to create richer representations than those obtained by using an atlas.

#### 2.4.1 Deep learning architecture and optimization

In this study, we used a self-supervised learning model called Champollion V0 (Laval et al., 2024), inspired by the Barlow Twins framework (Zbontar et al., 2021). This model utilizes a CNN with a 256-dimensional regional latent space to encode local sulcal morphology, and bring similar shapes together based on geometric patterns. To one cortical region corresponds one specific training and one latent space. In this work, we trained the model on the ACC region.

The architecture consists of a 12-layer convolutional backbone followed by a projection head made of two fully connected layers, with about 3,000,000 parameters in total (θ). The latent space corresponds to the output of the backbone, right before the projection head. Supplementary Figure S2 presents the model architecture.

Gaudin et al. (2024), then Laval et al. (2024) optimized the model’s ability to encode sulcal shape variability in its latent space using the linear probing strategy which is standard in SSL (i.e., by assessing linear evaluation of the latent space on sulcus-related classification or regression tasks). Champollion V0 (Laval et al., 2024) proved its ability to encode folding patterns of different natures, that is, topological (sulcus presence and sulcus interruption types) and non-topological (shape regression) patterns from 3 different cortical areas. The evaluations were performed on hand-labeled external databases that present a large domain gap with the training database: different machines, scanner resolutions, and populations. This ensures that Champollion V0 is to some extent robust to site effect. In particular, Champollion V0 exhibited high robustness to age, since one of the external databases consisted of teenagers (Cachia et al., 2016) while UkBioBank, the training database, contains subjects between 44 and 82 years old.

Among the three downstream tasks studied in Laval et al. (2024), one corresponds to the presence of the paracingulate sulcus in the ACC. The model achieves an ROC-AUC of 84% on the test set. Therefore, we are particularly confident in the quality of the representations in the ACC region used in this study. The other two tasks are described in detail in Laval et al. (2024) and provide insight into the patterns typically encoded. One task involves classifying four interruption types in the orbitofrontal cortex (Troiani et al., 2022), while the other involves a shape regression task using six continuous shape descriptors of the central sulcus, obtained using the Isomap algorithm (Z. Y. Sun et al., 2012).

#### 2.4.2 Training process

The training process, fully described in Laval et al. (2024), can be summarized as follows for a region of interest defined by a mask in the MNI space. For each masked skeleton from a batch of subjects, two augmented views are generated via a composition of random data augmentations T. The transformations used for the augmented views are small translations, fold depth trimming, cut-in (a parallelepiped box defines the voxels to be kept, setting those outside the box to zero), and cut-out (a parallelepiped box defines the voxels to be deleted, keeping only those outside the box). The two batches of augmented views $Y^{A}$ and $Y^{B}$ are then fed to a function $f_{\theta }$, here the CNN with trainable parameters θ, producing batches of embeddings $Z^{A}$ and $Z^{B}$ respectively. The Barlow Twins loss function is defined as (Zbontar et al., 2021, p. 2):

$${ℒ}_{BT}=\sum_{i}{\left(1-C_{ii}\right)}^{2}+\lambda \sum_{i}\sum_{j\neq i}C_{ij}^{2}$$ (1)

“where λ is a positive constant trading off the importance of the first and second terms of the loss, and where C is the cross-correlation matrix computed between the outputs of the two identical networks along the batch dimension” (Zbontar et al., 2021, p. 2):

$$C_{ij}=\frac{\sum_{b}z_{b,i}^{A}z_{b,j}^{B}}{\sqrt{\sum_{b}{\left(z_{b,i}^{A}\right)}^{2}}\sqrt{\sum_{b}{\left(z_{b,j}^{B}\right)}^{2}}}$$ (2)

“where b indexes batch samples and i,j index the vector dimension of the networks’ outputs. C is a square matrix with size the dimensionality of the network’s output, and with values comprised between -1 (i.e. perfect anti-correlation) and 1 (i.e. perfect correlation)” (Zbontar et al., 2021, p. 2).

It means that the neural network must maximize the similarity between the representations of the two augmented views of each subject, while minimizing redundancy between the dimensions of the representation space. The similarity term of the loss function forces invariances, for instance, relative to fold depth. The fact that fold depth has been shown to decrease with age (Kochunov et al., 2005) led Laval et al. (2024) to consider this invariance as important for age-independent folding patterns to emerge in the representation. All the configurations that vary only relative to fold depth should gather in the same domain of the representation space. The redundancy term in the loss function is used to prevent collapse. Indeed, using only the similarity term could lead to trivial solutions, such as projecting all the inputs to 0.

We trained two separate models (Champollion V0), one for the left ACC region and one for the right, to account for potential hemispheric differences in cortical folding patterns. Both are trained on the 36,000 subjects with white British ancestry (genetic discovery set) to ensure statistical robustness. The latent spaces of the trained networks can be seen as representations in which are encoded phenotypes derived from cortical folding morphology specific to the region of interest and the hemisphere under study. Once trained, the neural networks were used in inference mode to calculate the representations for both the 36,000 subjects used for training (genetic discovery set) and the 5,395 independent subjects with non-white British ancestry (genetic replication set). No genetic information is provided during training; only the mask of the sulcal skeleton relative to the region under study is used.

Following training, we observed an increase in loss of 0.6% between the training and replication datasets in the left hemisphere and a decrease of 1.1% in the right hemisphere. These marginal differences ensure the generalization of Champollion V0 to the replication dataset.

Then, we performed a Principal Component Analysis (PCA) on the 256-dimensional representation to reduce its dimensionality while preserving the most significant information. For each hemisphere, we retained only the minimal set of ranked PCA components explaining at least 99.9% of the variance. As a result, the left ACC region was encoded in 42 dimensions, while the right ACC region was encoded in 39 dimensions.

### 2.5 ACC without annotation

While the presence or absence of the PCS provides valuable insights, the latent space representation may encode richer information about the ACC folding pattern beyond this binary classification. Instead of limiting our analysis to a predefined folding pattern, we aimed to explore how genetic variations interact with the latent space representation. To achieve this, we investigated the underlying structure of the latent space by identifying meaningful directions that correlate with genetic data. Specifically, we applied a multivariate association study on the principal components derived from the latent space, allowing us to assess genetic influences across multiple dimensions simultaneously.

#### 2.5.1 Multivariate-GWAS on the representation space

The MOSTest approach, introduced by van der Meer et al. (2020), has been designed to identify associations between single nucleotide polymorphisms (SNPs) and a set of phenotypes. By combining summary statistics from independent GWASs for each phenotype, this method can reveal novel genetic variants. We briefly summarize it here.

Consider a set of k phenotypes. For a given SNP i, standard GWAS procedures give z-scores ${\text{z}}_{i}=\left\{z_{i1},z_{i2},\ldots ,z_{ik}\right\}$ ${\text{z}}_{i}=\left\{z_{i1},z_{i2},\ldots ,z_{ik}\right\}$, where $z_{ij}$ represents the z-score of the association between the SNP i and the phenotype j. For an SNP i, the multivariate test statistic is then defined as: $T_{i}={\text{z}}_{i}^{\text{T}}{\text{R}}^{-1}{\text{z}}_{i}$, where R is the correlation matrix between the k phenotypes, calculated in MOSTest using permuted genotypes. The test statistic is then compared to the null hypothesis, which is also estimated with the permuted genotypes.

In this study, instead of using cortical volumes, cortical thicknesses, and surfaces as van der Meer et al. (2020) did, the set of latent space dimensions (whose size depends on the region and the hemisphere) is used as input for each subject.

The following covariates are removed by pre-residualization; they are the same as the ones removed for the univariate GWASs on the PCS presence phenotype, see Appendix A (GWAS on the PCS phenotype): the 10 principal genetic components (UKB field: 22009), age (UKB field: 53), sex (UKB field: 31), ${\text{age}}^{2}$, and the acquisition site (UKB field: 54). Intracranial volume (ICV) and overall brain volume were not included as covariates, as some SNPs may influence cortical folding indirectly through their effects on brain or intracranial volume, and we aim to capture those associations as well.

### 2.6 Annotations of the results

First, we went back down to the level of the univariate GWAS results specific to each dimension of the representation spaces, to measure their estimated heritability, the lambda genomic control (GC), and the intercept with the LDSC software (Bulik-Sullivan et al., 2015), using the European reference panels (eur_w_ld_chr). This cannot be done with the MOSTest results, as the MOSTest z-scores are not signed.

Then, based on the MOSTest results, a genetic locus was considered validated if its lead SNP met three conditions: (1) it was associated in the discovery cohort with a p-value $<5\times 10^{-8}$ , the conventional GWAS threshold that accounts for the approximate number of independent SNPs genome-wide; (2) the same lead SNP was associated with the latent space in the replication cohort with a p-value <0.05​/​l , where l is the number of independent loci identified in the discovery set; (3) the distribution of p-values in the replication cohort was not inflated.

Following the protocol outlined by Watanabe et al. (2019) in the Functional Mapping and Annotation of GWAS (FUMA), a two-step pruning process was conducted. First, significant SNPs were pruned at a Linkage Desequilibrium (LD) threshold of $r^{2}=0.6$ to generate a list of independent significant SNPs. Next, these independent significant SNPs were further pruned at an LD threshold of $r^{2}=0.1$ to identify the independent lead SNPs.

A genomic locus was defined as the smallest contiguous region encompassing all SNPs (including both GWAS markers and those from the 1000 Genomes reference panel that pass the MAF threshold of 0.05) with an $r^{2}$ value greater than 0.1 with the lead SNPs. If the physical distance between adjacent loci was less than 250 kb, they were merged into a single locus.

The genomic inflation factor was estimated from Quantile-Quantile (QQ) plots, from FUMA.

Then, input SNPs were mapped to 18,842 protein-coding genes with a threshold for genome-wide significance defined at $2\times 10^{-6}$ (0.05​/​18842) after Bonferroni correction, with the Multi-marker Analysis of GenoMic Annotation (MAGMA) (de Leeuw et al., 2015). Additionally, MAGMA gene-set analysis was performed for curated gene sets and Gene Ontology (GO) terms obtained from the Molecular signatures Database (MsigDB), considering the default 17,008 gene sets, with a threshold for significance defined at $3\times 10^{-6}$ (0.05​/​17,008) after Bonferroni correction. A MAGMA tissue expression analysis was performed using data from BrainSpan, which includes 11 general developmental stages and 29 distinct brain sample ages. A MAGMA tissue type analysis was also performed using data from Genotype-Tissue Expression project (GTEx) v8 (Lonsdale et al., 2013).

### 2.7 Interpretation of genetics results in terms of folding patterns

Genetic associations served as a framework to identify cortical folding patterns within the latent space. We extracted the corresponding genotyping data for lead SNPs identified by FUMA as associated with these latent space representations, determining the exact alleles for each subject.

To validate our approach, we analyzed correlations between the z-scores of all SNPs significantly associated with the set of dimensions ($p<5\times 10^{-8}$ ). This analysis revealed a linkage disequilibrium map, as illustrated in the Supplementary Figures S8 and S15. This correlation map enables us to assess whether SNPs strongly associated with the set of dimensions exhibit consistent univariate GWAS patterns and whether a lead SNP reliably represents its entire block of dimensions.

**Fig. 3.**
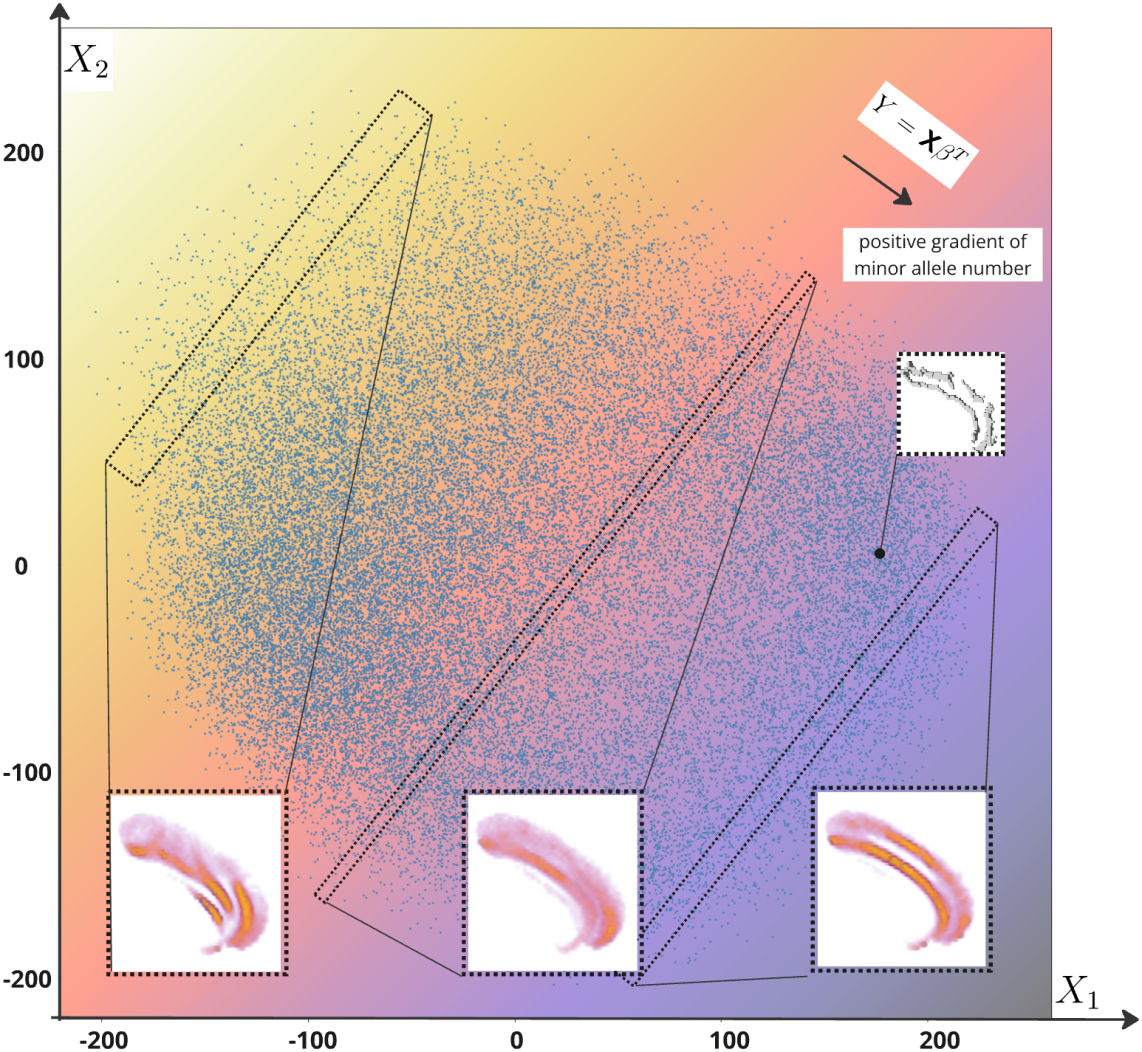
From a lead SNP to a variation of folding pattern. To clarify the interpretation method, we plot only the two principal components $X_{1}$ and $X_{2}$ from the latent space representing the left ACC region. In this space, we perform a least-squares regression to predict a simulated variable Y, which represents the genotype as the number of minor alleles (ranging from 0 to 2) for a specific SNP of interest. The color gradient in the plot indicates the transition from 0 to 2 minor alleles. Additionally, we group individuals into blocks of 200 subjects along this gradient. For each block, we compute the average sulcal shape (in orange), which is also visualized. Finally, we highlight a single subject as an example (in grey). Note that, in our actual interpretation, we are not using only the first two components of a PCA but all the components needed to explain 99.9% of the variance.

We applied an Ordinary Least Squares (OLS) regression to the pre-residualized embedding (the input for MOSTest) to evaluate how well the n -dimensional embedding could predict genetic variation for a given significative SNP. Following the same additive hypothesis as in GWAS, we ranked individuals based on their model-predicted number of minor alleles (Y). $Y=X\beta^{T}+\epsilon$ , with $\text{X}=\left(X_{1},X_{2},\ldots ,X_{n}\right)$ the n dimensions of the latent space, $\beta =\left(\beta_{1},\beta_{2},\ldots ,\beta_{n}\right)$ coefficients to determine, and ϵ the error term. We grouped subjects into batches of 200 subjects based on this ranking to compute batch-wise averages (Fig. 3). In the final plot, voxel colors range from purple (present in at least 5% of subjects) to yellow (present in more than 35%). The moving average highlights shared voxels among subjects, revealing factors that bring them together in the representation space.

This process provides a means of making information emerge from the representation space by following the guidance of genetic association.

## 3 Results

### 3.1 ACC and human PCS annotation

#### 3.1.1 Generalization in the UK BioBank

Using Champollion V0 trained on 36,000 subjects with white British ancestry, we obtained a 256-dimensional latent space for each hemisphere, representing the ACC region folding variability. Each subject from the ACC dataset (with an annotated PCS) was then projected into this latent space via model inference.

Using the projection of the ACC dataset subjects into the latent space, we trained a support vector machine (SVM) to predict the presence or absence of a PCS. To optimize the SVM, we evaluated its performance with a cross-validation on 10 stratified folds (label, acquisition site and sex) (n=273 ), achieving an (ROC AUC) of 83.2% for the left hemisphere and 86.6% for the right hemisphere. The SVM parameters are: a linear kernel, a balanced class weight, and C=0.01 for a better trade-off between the training error and the margin. This model identified a hyperplane that separates subjects with a PCS from those without it in the representation of the ACC dedicated database.

To maximize statistical power, we trained a second classification model with the same hyperparameters using the entire ACC dataset (n=341 , of which 225 annotated with the PCS in the left hemisphere and 152 with the PCS in the right hemisphere). This classifier was then applied to the UK Biobank (UKB) dataset (n=41,395 ), which lacks manual annotation of the paracingulate sulcus, allowing us to predict the presence of a PCS in this larger population in terms of probability. The PCS phenotype can be viewed as a row vector of length n, with each element representing the predicted probability of PCS presence for a subject.

**Fig. 4.**
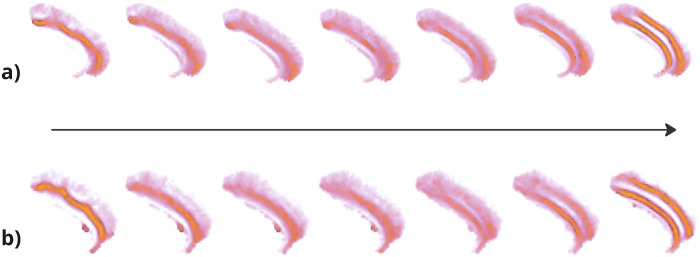
Moving average visualization of UKB subjects automatically classified based on their probability of having a paracingulate sulcus (PCS). Each average is composed of 200 subjects. (a) Left hemisphere results, with subjects sorted in ascending order of predicted probability. (b) Right hemisphere results, mirrored to match the left hemisphere configuration for better comparison. As could be expected, the moving average on the right, corresponding to a higher probability of having a PCS, presents a long PCS.

To validate the classifier in the UKB database, both quantitative and qualitative methods were used. For the quantitative approach, we used a never-seen-before test base for the classification model, consisting of 100 right and 100 left hemispheres from 100 UKB subjects. Besides providing a test set, the manual annotation of the UKB subjects was made on an age-stratified set. Indeed, we first divided the 42,433 subjects into 10 deciles according to age. Then, we randomly picked up 25 females among the first decile, 25 males among the first decile (average age 51 years), 25 females among the last decile, and 25 males among the last decile (average age 77 years), resulting in 100 subjects. Six independent raters annotated the 100 subjects according to the nomenclature used in Cachia et al. (2016). The ground truth was defined as the most frequently used label among the six ratings. In cases of a tie (seven out of 200 hemispheres), it was agreed that PCS was present. There is no difference in pattern distribution with respect to extreme ages (Supplementary Fig. S3). The ROC AUC is 85.9% for the left hemisphere and 85.6% for the right hemisphere on this test set (UKB), while it was previously equal to 83.2% for the left and 86.6% for the right with the cross-validation on 10 stratified folds (ACC dataset). There is a slight loss of ROC AUC for the right hemisphere, but this remains a convincing generalization. For the qualitative approach, we ordered subjects according to the yielded probability of PCS presence. Based on this ranking, we calculated the skeleton averages in the ACC mask for groups of 200 consecutive subjects located at regular intervals between 0 and 1. In the final plot, the voxel colors range from purple (present in at least 5% of the subjects) to yellow (present in more than 35%). It is the same average as the one presented in Figure 3. The moving average highlights shared voxels among subjects. The results highlight that in each hemisphere, the average of subjects with a high probability of PCS shows a clear “double parallel” pattern, while the average of subjects with a low probability of PCS only highlights CS (see Fig. 4).

#### 3.1.2 GWAS on the PCS phenotype

**Appendix Figure A1.**
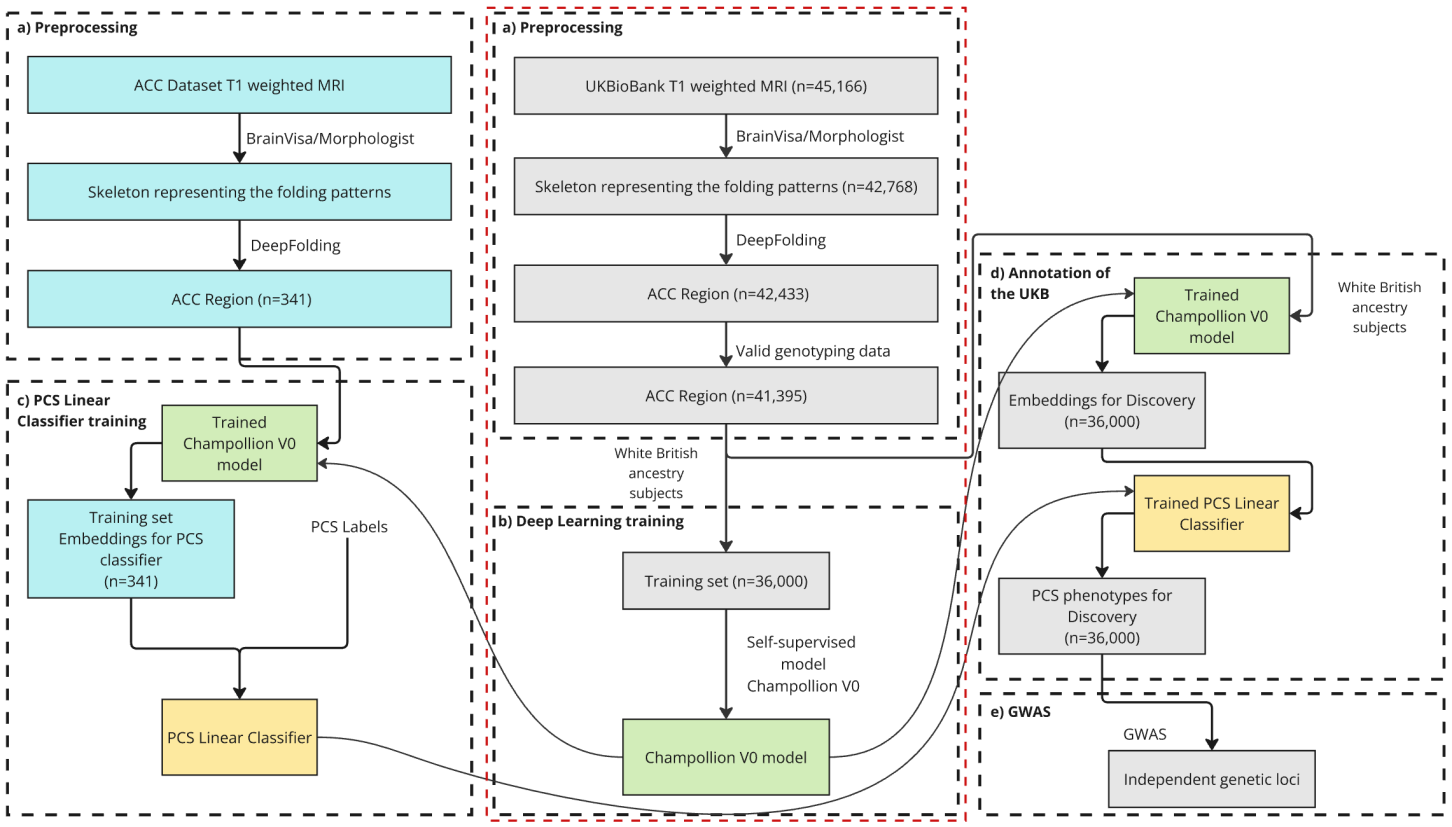
Overview of PCS annotation-based method for one hemisphere. (a) and (b) (framed with red dots) are rigorously the same steps as those in Figure 2, to show that the same deterministic model is used to encode the ACC region. (c) The AD subjects (blue) are encoded in the representation space, and a linear classifier is trained to predict the presence of PCS based on the manually annotation. (d) The UKB subjects are encoded in the representation space using the same Champollion V0 model. The linear classifier trained on the ACC dataset subjects yields the probability of having the PCS for the 36,000 UKB subjects. (e) A GWAS is made on the PCS phenotype on the 36,000 UKB subjects.

The method relying on the human PCS annotation is summarized in Appendix A, with a helpful illustration (Appendix Fig. A1).

#### 3.1.3 Genetic association with the PCS annotation

Using the approach based on PCS annotation (in terms of probability) for the right hemisphere on the UKB subjects (discovery cohort, n=36,000 ), no SNP reached genome-wide significance at the conventional threshold of ($5\times 10^{-8}$ ). As highlighted in Fig. 4b), the automatic classification worked well, demonstrating very long sulci parallel to the CS on subjects best predicted as having the PCS, with a progressively less prominent second sulcus as the predictive probability of PCS decreases.

For the left hemisphere as well, no SNP p-value is strictly below genome-wide significance at the conventional threshold of ($5\times 10^{-8}$ ). Note that 8 SNPs, based on the locus 17q11.2, have a $p_{d}=5\times 10^{-8}$ .

### 3.2 ACC without annotation

#### 3.2.1 Right ACC region

To investigate heritability at the level of each latent dimension encoding for the right ACC region, we computed heritability estimates using the LDSC software (Bulik-Sullivan et al., 2015). As shown in Supplementary Table S1, the first two principal components of the latent space exhibit the highest heritability (7.8%). Beyond the 22th dimension, no significant heritable signal is detected.

In the discovery phase, with our annotation-free approach directly based on the representation space provided by Champollion V0, we identified four genomic loci associated with the folding pattern of the right ACC region (summarized in Supplementary Table S2, with the regional plots in Supplementary Figs. S9–S12), resulting in three genes after the MAGMA analysis (Supplementary Table S3). However, none of the loci identified in the discovery set achieved replication in this study, as the associations for the lead SNPs were not statistically robust (see the $p_{r}$ column >0.05​/​4 ). Notably, the genomic locus 5q14.3, represented by the lead SNP rs309588, showed a nominally significant association in the replication phase ($p_{r}=0.05$ ).

In the discovery set, no gene sets were significantly associated with the right ACC region. Likewise, no significant associations were observed for developmental stages or tissue types.

#### 3.2.2 Left ACC region

To investigate heritability of the left ACC region at the level of each latent dimension, we computed heritability estimates using the LDSC software. As shown in Supplementary Table S4, the first and the fifth principal components of the latent space exhibit the highest heritability (12.6% and 7.9%), which is more than in the right ACC region. Furthermore, beyond the 25th dimension, no significant heritable signal is detected. Therefore, more dimensions play a role in the multivariate association for the left ACC than for the right ACC.

**Fig. 5.**
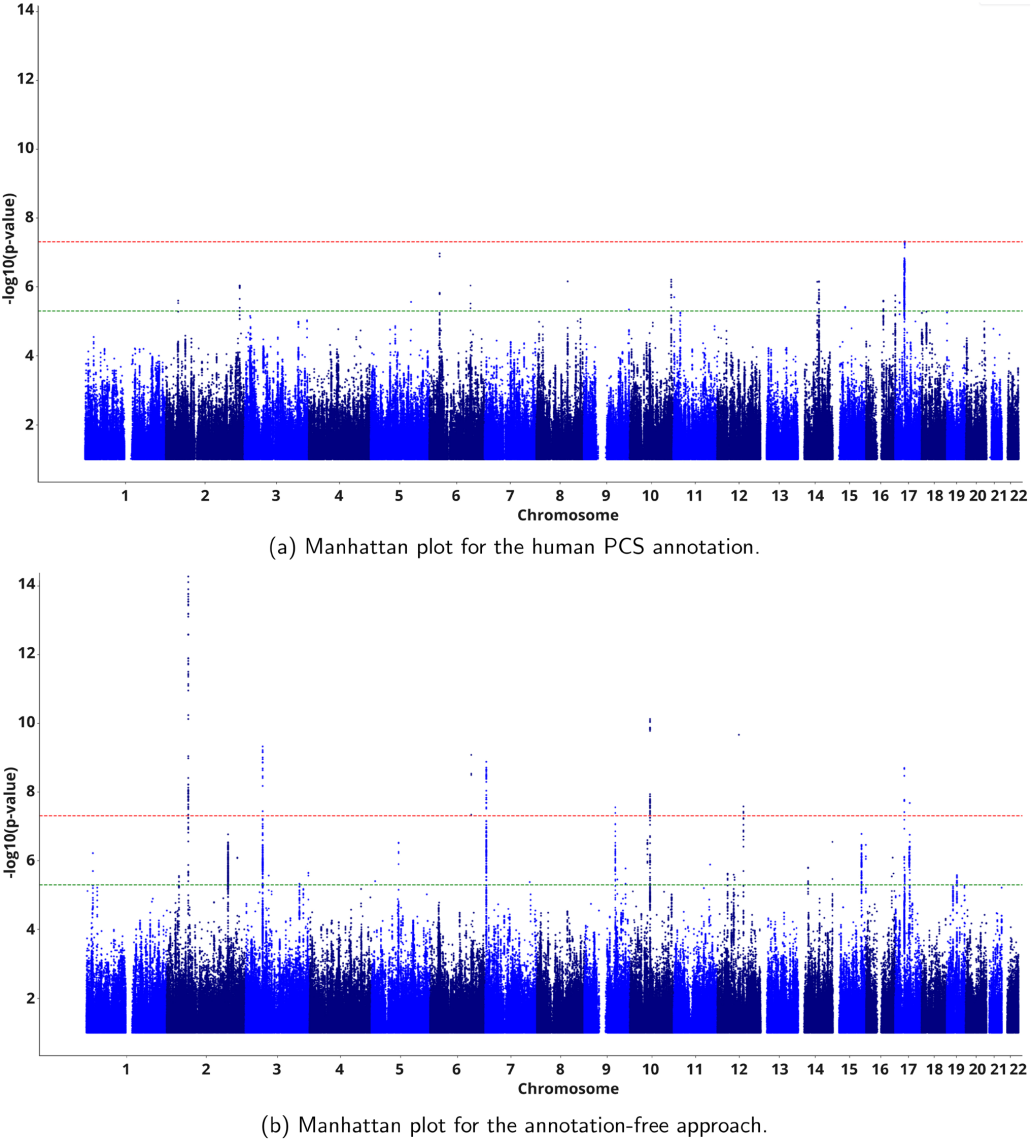
Manhattan plots showing significance of each variant’s association with the left ACC region, for (a) the human PCS annotation and (b) the annotation-free approaches. Each dot represents an SNP, with SNPs ordered on the x-axis according to their genomic position. The red line marks the SNP significance threshold $5\times 10^{-8}$ . The green line marks the SNP suggestive threshold $5\times 10^{-6}$ .

With our annotation-free approach directly based on the representation space provided by Champollion V0, and while only four loci were found in the right ACC region in the discovery phase, 10 loci were identified as associated with the left ACC region (summarized in Table 1, with the Manhattan plot in Fig. 5b and regional plots in Supplementary Figs. S16–S20), resulting in 10 genes (Table 2). Notably, the gene set analysis revealed five functions directly related to neurodevelopment and brain growth, when looking at the most significant gene-set associations based on their p-values (Table 3). Axon development exhibited a significant association ($p=1\times 10^{-6}$ ) compared to the $3\times 10^{-6}$ threshold (0.05​/​17,008), while neuron projection guidance ($p=7\times 10^{-6}$ ), cell part morphogenesis ($p=1\times 10^{-5}$ ), developmental cell growth ($p=2\times 10^{-5}$ ), and cellular component morphogenesis ($p=2\times 10^{-5}$ ) also showed suggestive associations.

**Table 1.** Genomic loci associated to the left ACC region (n = 36,000).

Lead SNP	A1	Chr	BP	$p_{d}$	$p_{r}$	IndSigSNPs
rs2009778	C	2	65977863	$6\times 10^{-15}$	0.004	rs2009778; rs10176894; rs3815606
rs6445528	A	3	52572447	$5\times 10^{-10}$	0.2	rs6445528
rs58321169	C	6	126868567	$9\times 10^{-10}$	0.7	rs58321169
rs798536	G	7	2766383	$2\times 10^{-9}$	0.07	rs798536
rs34739795	C	9	95934431	$3\times 10^{-8}$	0.6	rs34739795
rs10740020	C	10	62052544	$8\times 10^{-11}$	0.3	rs10740020; rs7073658
rs79487293	C	12	65905126	$3\times 10^{-10}$	0.5	rs79487293
rs4842267	T	12	80019427	$3\times 10^{-8}$	0.02	rs4842267
rs11080107	T	17	27938424	$2\times 10^{-9}$	0.2	rs11080107
rs12951057	C	17	44794558	$2\times 10^{-8}$	0.09	rs12951057

The tables show loci information, including the rsID of the lead SNP, allele 1 (A1), chromosome (Chr), base pair (BP) position of lead SNP on hc19, p-value for the discovery cohort (n = 36,000) ($p_{d}$), for the replication cohort (n = 5,395) ($p_{r}$), and independent significant SNPs (IndSigSNPs), from FUMA.

**Table 2.** Mapped genes associated to the left ACC region.

GENE	CHR	START	STOP	Z	p
NT5DC2	3	52558386	52569070	5.58	$2\times 10^{-8}$
STAB1	3	52529354	52558511	5.44	$3\times 10^{-8}$
ANK3	10	61786056	62493248	5.26	$8\times 10^{-8}$
CORO6	17	27941774	27949925	5.24	$8\times 10^{-8}$
WNK2	9	95947198	96082854	4.96	$4\times 10^{-7}$
AMZ1	7	2719156	2815134	4.87	$6\times 10^{-7}$
GNA12	7	2767746	2883958	4.82	$8\times 10^{-7}$
SMIM4	3	52568029	52613253	4.73	$1\times 10^{-6}$
SPCS1	3	52738971	52742182	4.63	$2\times 10^{-6}$
CALCRL	2	188207856	188313187	4.63	$2\times 10^{-6}$

This table lists the mapped genes locations regarding the p-values below the threshold $2\times 10^{-6}$, n = 36,000, from MAGMA.

**Table 3.** Gene set associated to the left ACC region.

NAME	$N_{\text{GENES}}$	β	$\beta_{\text{STD}}$	SE	p
GOBP Axon Development	441	0.20	0.03	0.04	$1\times 10^{-6}$
GOBP Neuron Projection Guidance	210	0.28	0.03	0.06	$7\times 10^{-6}$
GOBP Cell Part Morphogenesis	612	0.16	0.03	0.04	$1\times 10^{-5}$
GOBP Developmental Cell Growth	207	0.25	0.03	0.06	$2\times 10^{-5}$
GOBP Cellular Component Morphogenesis	720	0.14	0.03	0.03	$2\times 10^{-5}$

This table summarizes the top 5 gene set enrichment analysis results regarding the p-values, including the number of genes per set ($N_{\text{GENES}}$), beta coefficients (β and $\beta_{\text{STD}}$), standard error (SE), n = 36,000, from MAGMA.

The analysis of gene expression in all developmental stages (Supplementary Table S5; Supplementary Fig. S22) highlighted strong associations with prenatal stages (p<0.05 ), including early prenatal (p=0.002 ), early mid-prenatal (p=0.0002 ), late mid-prenatal (p=0.002 ), and late prenatal (p=0.05 ).

In the replication set (see the column $p_{r}$), only the locus 2p14, represented by the lead SNP rs2009778 ($p_{r}=0.004$ ), could be robustly validated, regarding the threshold 0.05​/​10 . However, loci 7p22.3 (lead SNP rs798536, $p_{r}=0.07$ ), 12q21.2 (lead SNP rs4842267, $p_{r}=0.02$ ), and 17q21.31 (lead SNP rs12951057, $p_{r}=0.09$ ) remain of interest for further investigation.

#### 3.2.3 Interpretation in terms of folding pattern variations

**Fig. 6.**
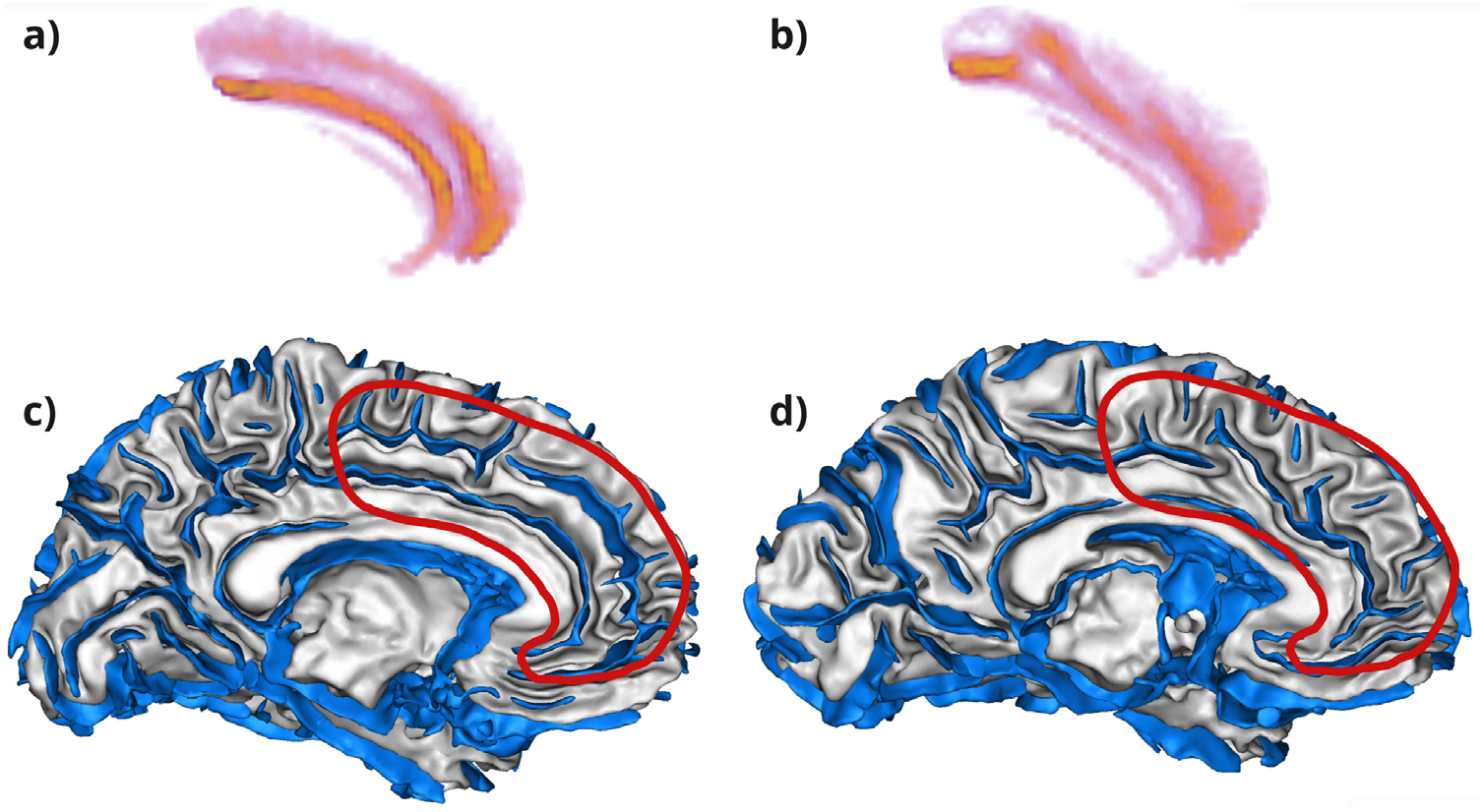
Averages of extreme subject for the lead SNP rs2009778 ($p_{d}=6\times 10^{-15}$ , $p_{r}=0.004$ , locus 2p14). (a) and (b) The two extreme averages, calculated on 200 subjects each, corresponding to the 200 most extreme subjects indicated by the linear model trained to determine the number of major allele for the lead SNP rs2009778 (on the locus 2p14), for the left ACC region. (c) and (d) Represent examples of subjects with the corresponding pattern found above, displayed together with the white matter mesh (in grey) and the sulci (in blue).

Regarding interpretation of the folding pattern variations encoded by the latent space direction associated to the replicated locus 2p14 (Fig. 6), we observe the presence of the CS and the PCS in the average computed on one side, looking like the classical double-parallel configuration while we observe only an interrupted CS and no PCS on the other side.

Moreover, an analysis of the pattern variations associated with SNPs rs2009778, rs798536, rs4842267, and rs12951057 (Supplementary Fig. S21) reveals that the location of common voxels (highlighted in yellow) is linked to the presence of a PCS. However, the length and precise positioning of the PCS vary across averages. Additionally, interruptions in the CS appear to play a crucial role in the observed variations.

#### 3.2.4 Comparison between hemispheres

Regarding the discovery phase, two loci are shared between both hemispheres. The first is 2p14, represented by the lead SNP rs10168499 for the right ACC region and rs2009778 for the left. The second is 3p21.1, indicated by the lead SNP rs6445528 for the left hemisphere and rs7636227 for the right. Consequently, the same two genes, STAB1 and NT5DC2, are found to be associated with the folding pattern of the ACC in both hemispheres.

## 4 Discussion

In this paper, we proposed an innovative approach to study the associations between genetics and folding patterns, which overcomes the limitations of the current cortical folding visions. On the one hand, the sulci atlases used in the field, along with the few proposed folding pattern shapes in the literature, allow only a partial exploration of the neuro-developmental genesis. On the other hand, the representations that emerge from our foundation model are likely to bring to light cortical folding patterns under genetic influence that have yet gone unnoticed. It must be stressed that this article explores the representation space only through the prism of genetically influenced folding patterns. Other interesting folding patterns could emerge in the future using clinical or environmental information.

To the best of our knowledge, performing a multivariate genetics analysis of a cortical folding representation inferred by a deep learning model has not been done before. Until now, attempts to explore the genetics of folding patterns have mainly relied on supervised models derived from BrainVISA software, which automatically identifies sulci from a nomenclature that does not contain the notion of PCS (Fish et al., 2017; Kochunov et al., 2010; Le Guen, Leroy, et al., 2018; Le Guen et al., 2019; McKay et al., 2013; Pizzagalli et al., 2020; B. B. Sun et al., 2022) and would not allow one to study additional folding patterns beyond the reach of this nomenclature. The genetic architecture of sulcal depth (Van Der Meer et al., 2021) and the heritability of cortical surface curvature (Schmitt et al., 2021) have also been studied, but linking these results with folding pattern variability is not straightforward.

Another strategy likely to bring out an understanding of the genetics associated with folding pattern variability is the one that detects cortical surface sulcal pits (Auzias et al., 2015), which are significant local depth extrema. This strategy could indeed quantify the influence of genetics on how neighboring pits are connected or not by cortical folds (Le Guen, Auzias, et al., 2018). However, this approach describes local cortical folding variants less exhaustively than ours.

To put our findings into perspective, we compared the lead SNPs we identified with the GWAS catalog, evaluating their relevance in previous studies. For the right ACC association, the loci 1p36.13 (lead SNP rs2854108, $p_{d}=2\times 10^{-10}$ ), 2p14 (lead SNP rs10168499, $p_{d}=2\times 10^{-10}$ ), 3p21.1 (lead SNP rs7636227, $p_{d}=5\times 10^{-9}$ ), and 5q14.3 (lead SNP rs309588, $p_{d}=6\times 10^{-11}$ ) have been previously associated with cortical thickness, surface area (Shadrin et al., 2021), and their combination (Van der Meer et al., 2022). Also, the locus 3p21.1 (lead SNP rs7636227, $p_{d}=5\times 10^{-9}$ ) has shown associations with verbal learning (Lahti et al., 2022) and bipolar disorder (Li et al., 2021; Y. Wu et al., 2020).

For the left ACC association, all of the lead SNPs and related loci are included in Shadrin et al. (2021) and Van der Meer et al. (2022) studies as well, which is coherent because they both worked on cortical surface area and cortical thickness, two metrics that are related to brain folding. Indeed, the cortex is thicker in the crown of the gyri than at the bottom of the sulci (Wagstyl et al., 2018). The locus 17q21.31 (lead SNP rs12951057, $p_{d}=2\times 10^{-8}$ ) includes 19 candidate genes for cortical expansion phenotypes, curvature phenotypes, and neurite density (Warrier et al., 2023).

At the gene level, both STAB1 and ANK3 have been associated with bipolar disorder and schizophrenia (Holmgren et al., 2022). Ankyrin-G, the protein encoded by the ANK3 gene, plays a key role in anchoring cell adhesion molecules at the nodes of Ranvier and the axon initial segments (Kordeli et al., 1995; Yoon et al., 2022). WNK2 has been implicated in neurite outgrowth and differentiation (Shimizu & Shibuya, 2022), while SMIM4 has been linked to anxiety disorders, particularly in the anterior cingulate cortex (Friligkou et al., 2024). NT5DC2 has been associated with schizophrenia, bipolar disorder, in relation to abnormal activity of dopamine in the brain (Ripke et al., 2013; van Hulzen et al., 2017; Yamaguchi et al., 2024).

The fact that the genes we highlighted using only the ACC sulcal skeleton are also associated with other phenotypes describing cortical anatomy leads to an important question. The traditional paradigm for quantifying phenotypes, such as cortical thickness, relies on spatial normalization to produce comparable measurements across subjects. This spatial normalization consists of aligning the main cortical sulci across subjects as much as possible when matching cortical surfaces. However, in regions with a significant amount of folding alternative patterns, it is impossible to get rid of the folding variability. Consequently, since the thickness of the cortex varies massively between the bottom of the folds and the top of the gyri (Wagstyl et al., 2018), it is likely that some of the reported genetic associations with cortical thickness are actually associations with folding patterns. Moreover, this distinction is of the utmost importance when interpreting the findings, as most of the folding dynamics leading to patterns occur in utero during neurogenesis, while the variation of cortical thickness may also result from phenomena occurring much later, during cortical maturation, or as a result of neurodegenerative phenomena.

At the present stage of our understanding of the phenomena underlying folding dynamics, it is hard to imagine why a genetic variant might favor one of the two folding averages highlighted in our interpretations. Nevertheless, we can sketch out a few hypotheses linked to the proposed mechanical models (Tallinen et al., 2016). In one of the averages obtained after the association with the lead SNP rs2009778 representing the locus 2p14 ($p_{r}=0.004$ ), a fusion between the calloso-marginal posterior fissure (F.C.M.post) and the calloso-marginal anterior fissure (F.C.M.ant) is observed, leading to an uninterrupted CS. To the extent that the CS develops earlier than the eventual PCS (Chi et al., 1977), this fusion could favor the emergence of the PCS because of minimal interference with its folding trajectory (Fig. 6a). In return, in the alternative average, the F.C.M.post seems to go under the F.C.M.ant, which could disturb the formation of a PCS (Fig. 6b). Nevertheless, even among the 200 subjects composing each average, a lot of variability can be found.

Emerging deep-learning-based approaches are transforming the way we study human brain structures, particularly in the context of genetic associations. One such method, proposed by Patel et al. (2024), combines unsupervised deep learning representations with genetic association analyses. Their interpretation approach relies on a perturbation-based decoder. Using T1 and T2-FLAIR MRI data, Patel et al. (2024) successfully constructed a latent space representation of the entire brain structure. They then applied targeted perturbations to the components within this latent space that are significantly associated with specific SNPs. By decoding these perturbations, they visualized which brain regions were altered compared to the original, unmodified reconstruction. This method follows a top-down approach, meaning it starts with a representation of the whole brain and then attempts to localize genetic effects. However, a potential limitation of this strategy is that if the combination of many components produces the genetic signal, meaningful association locations may be missed. In contrast to Patel et al. (2024), our study employs a bottom-up approach where the structures of distant regions cannot influence the results of the regions under study.

Despite the strengths of our approach, several limitations must be acknowledged.

The X chromosome was not included in this study.

For the annotation-free framework, the number of latent dimensions differed between the left ACC (42) and the right ACC (39), which could influence the multivariate association tests. Higher-dimensional representations may capture more information about a phenotype but can also dilute the statistical signals of interest. More importantly, the number of dimensions was chosen to retain 99.9 % of the variance in the representation spaces, and not because they were heritable. We can clearly see that a heritability threshold is conceivable to choose the number of dimensions to keep, given the Supplementary Tables S1 and S4. In our case, the genetic difference measured between the two hemispheres must be related to the higher heritability of the left ACC compared to the right ACC region (12.6 % against 7.8 % for the first dimension of the representation space after PCA).

Another important consideration is the analysis of representation spaces from a genetic perspective. To address this, we used the MOSTest to compare the results of univariate GWAS across dimensions. The MOSTest identified associations resulting from more diffuse genetic signals distributed across multiple dimensions. This contrasts with the minP approach, which prioritizes signals that are strongly localized to a single dimension. As a result, minP identified three loci in the left ACC. The three loci differed from those detected by MOSTest (5q14.3 with the lead SNP rs310528 $p=1\times 10^{-9}$ , 11p14.1 with the lead SNP rs560061006 $p=2\times 10^{-8}$ , 15q25.3 with the lead SNP rs2679089 $p=3\times 10^{-11}$ ). No loci were found in the right ACC with the minP approach.

The lack of replication of the MOSTest associations may be due to sample size and differences in genetic ancestry within the replication set (Supplementary Figs. S7 and S14). A larger, less diverse, and independent dataset is necessary to confirm our findings. Note that the inflation factor observed in the QQ plots, (lambda GC equal to 1.16 for left ACC and 1.12 for the right ACC; Supplementary Figs. S6 and S13) is acceptable considering the number of subjects in the discovery cohort. In parallel, we also incorporated the 40 principal genetic components provided by UKB, while leaving all other parameters unchanged, as recommended in Smith et al. (2021), to account for population structure. Notably, their inclusion had almost no impact on our findings: the lambda GC for all MOSTest summary statistics decreased only slightly (−0.008 ; see Supplementary Fig. S23), and the association results remained unchanged. The same lead SNPs were identified (10 in the left ACC and 4 in the right ACC) in the discovery cohort, with p-values comparable to those obtained when using 10 genetic PCs as covariates, and replication did not improve.

And it is still too early to validate our interpretation in terms of folding. We assumed that a direction of latent space associated with a leader SNP describes a transition between two folding patterns, with the clearest shapes corresponding to the subjects positioned at the ends. Interestingly, in the case of the reproduced SNP, one of the two folding patterns corresponds to the double-parallel configuration, that is, to the presence of the PCS (Fig. 6). Insofar as this pattern has been repeatedly associated with psychoses (Bersani et al., 2014; Fornito et al., 2008, 2009; Le Provost et al., 2003; S.-C. J. Wu et al., 2024; Yücel et al., 2002), its identification with our genetic-based exploratory approach is of interest, even if it is the principal axis of variation in the representation space, other features such as curvature or branching patterns must contribute meaningfully. It should also be noted that other genes detected by our approach have already been associated with schizophrenia and bipolar disorder. Therefore, there is a strong presumption that particularities of the ACC folding patterns could be associated with psychiatric pathologies, but a more sophisticated model of cortical folding shapes will be needed to clarify this. Our results are a first step in this direction.

An essential aspect of this framework is its applicability across a set of sulcal regions covering the whole brain. This will allow a granular investigation of the genetic determinants of cortical folding. Ultimately, this approach could yield a comprehensive map of genetic associations and heritability estimates for each sulcal region, paving the way for local genetic correlation with diseases.

## Supplementary Material

Supplementary Material

## Figures and Tables

**Appendix Table A1.** Inter-rater variability (kappa metric).

	A	B	C	D	E	F
A	1.00	0.68	0.65	0.69	0.70	0.58
B	0.68	1.00	0.64	0.70	0.75	0.57
C	0.65	0.64	1.00	0.64	0.62	0.59
D	0.69	0.70	0.64	1.00	0.68	0.43
E	0.70	0.75	0.62	0.68	1.00	0.47
F	0.58	0.57	0.59	0.43	0.47	1.00

## Data Availability

Champollion V0 codes can be found on the following git repository: https://github.com/neurospin/champollion_V0. The analysis of the latent representations can be found on the following git repository: https://github.com/neurospin-projects/2024_adufournet_sulcus_genetics. GWAS summary statistics are uploaded at https://doi.org/10.5281/zenodo.17043961.

## Footnotes

†
https://brainvisa.info/web/morphologist.html

‡
https://github.com/neurospin/deep_folding

## Appendix A

### GWAS on the PCS Phenotype

We treated the previously obtained PCS probabilistic annotation of each UKB subject as phenotypic data. Using the general linear model implemented in PLINK2 (version 17-02-22), we conducted a GWAS. The GWAS analysis incorporated the following covariates: the first 10 principal genetic components (UKB field: 22009), age (UKB field: 53), sex (UKB field: 31), ${\text{age}}^{2}$, and the acquisition site (UKB field: 54). A significance threshold of ($5\times 10^{-8}$ ) was applied to identify statistically significant associations.

### Inter-rater variability in the manual annotation of the 100 UKB subjects

When doing the manual annotation task on the 100 subjects, we evaluated the inter-rater variability, with the rater going from A to F, with the kappa metric presented in Appendix Table A1.

As we can see, there is an important variability when labeling the PCS, due to the presence of the intracingulate sulcus that can be interpreted as a cingulate sulcus (less than 1 case out of 10), or due to a potential PCS length really close to 20 mm (Supplementary Figs. S4 and S5).
