# Supplementary material for: A self-supervised learning framework for discovering cortical folding patterns under genetic influence: Application to the Anterior Cingulate Cortex
Source: Imaging Neurosci (Camb). 2025 Nov 5;3:IMAG.a.987. doi: 10.1162/IMAG.a.987 (PMC12592371; doi:10.1162/IMAG.a.987)
Supplement: Supplementary Material [file IMAG.a.987_supp.pdf]

Supplemental Material

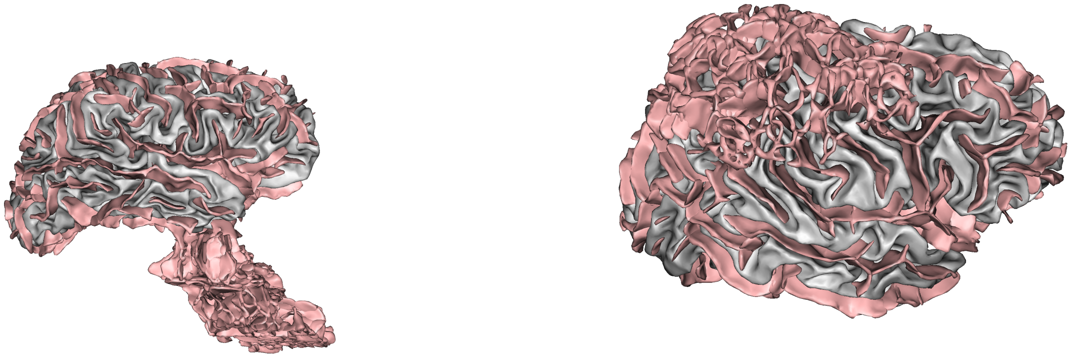

(a) Bad segmentation. (b) Too many voxels in the sulcal skeleton.

Figure S1: Examples of hemispheres excluded during quality control. The hemisphere on the left has a bad segmentation, while the hemisphere on the right has more than 1.2 times the number of voxels compared to the last decile in their sulcal skeleton.

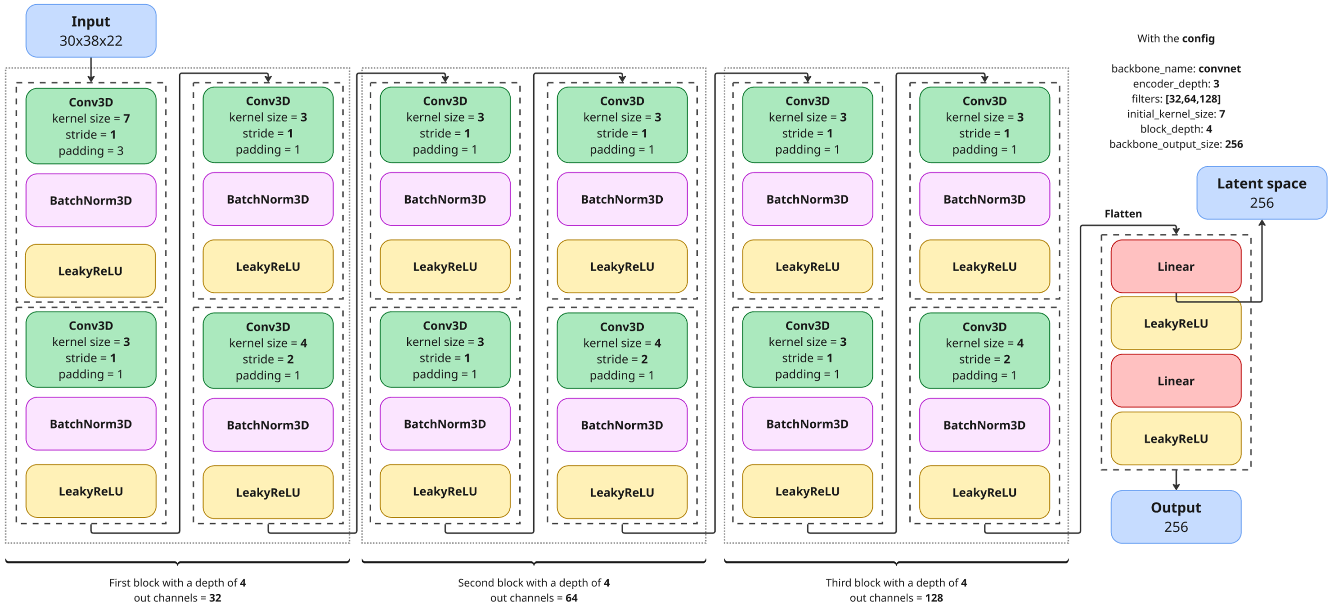

Figure S2: Details of Champollion V0 architecture.

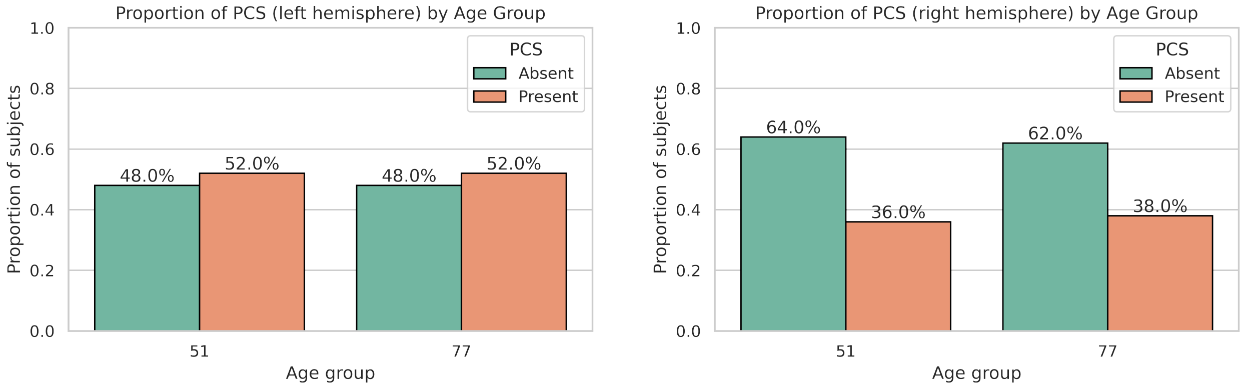

(a) Left hemisphere.

(b) Right hemisphere.

Figure S3: Proportion of PCS by age, for both hemispheres. The key point of this graph is the equal proportion of PCS in both age groups (50.7 ± 1.5 years and 76.5 ± 1.5 years), for a given hemisphere. The proportion of PCS is also greater in the left hemisphere than in the right. From the subset of 100 UKB subjects (50% male) who were manually annotated for PCS presence.

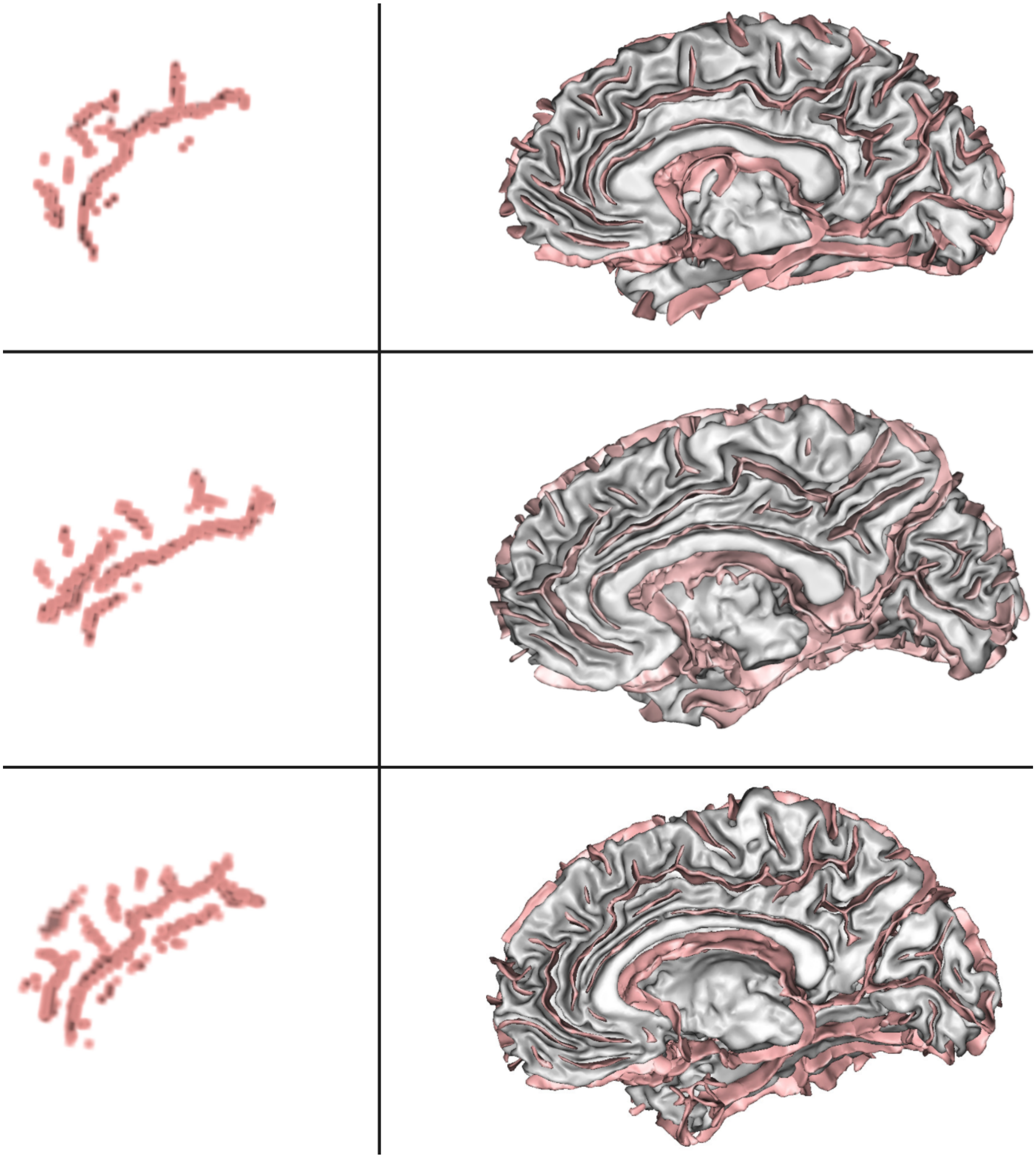

Figure S4: Examples of boundary cases, where the classifier tends to struggle. On the left, the binary entry for Champollion V0, extracted from the sulcal skeleton visible on the right, on the white mesh. There is an overlap that may be too short to be considered a PCS in each right hemisphere shown here (less than 20 mm).

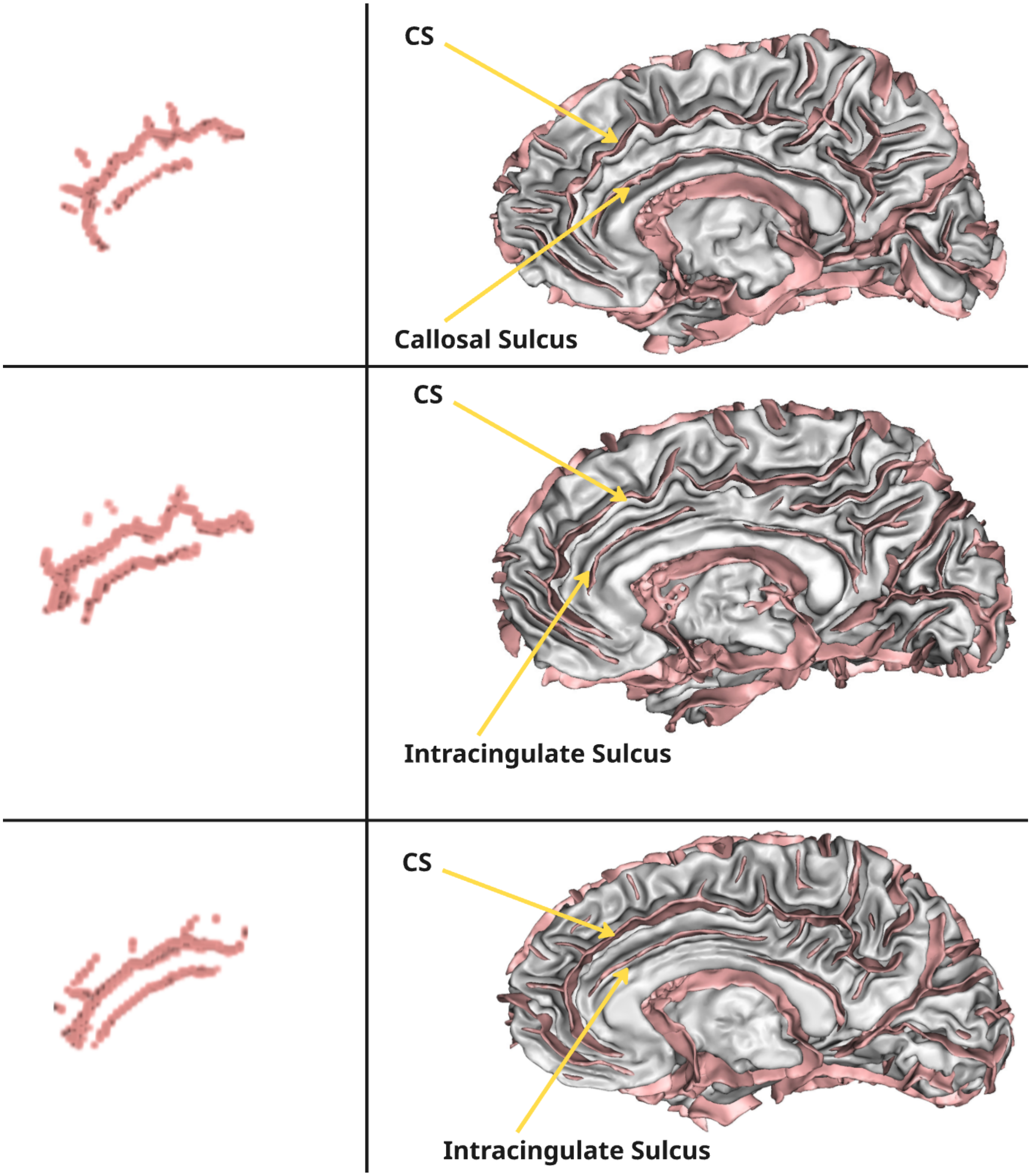

Figure S5: Examples of cases where the input is misclassified by the model. On the left, the binary input for Champollion V0, extracted from the sulcal skeleton seen on the right, on the white mesh. Note the presence of an intracingulate sulcus located between the callosal sulcus and the cingulate sulcus, which could be interpreted as a cingulate sulcus, whereas the cingulate sulcus plays the role of a para-cingulate sulcus for the classification model.

0.1 Right ACC Figures

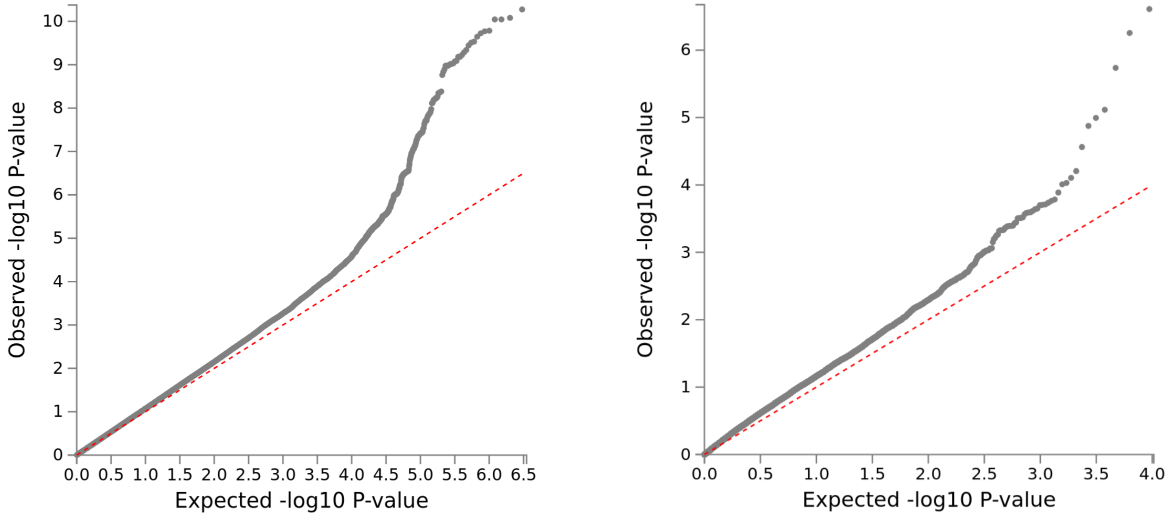

(a) SNP based QQ plot. (b) Gene based QQ plot.

Figure S6: QQ plots for the Right ACC Region (from FUMA).

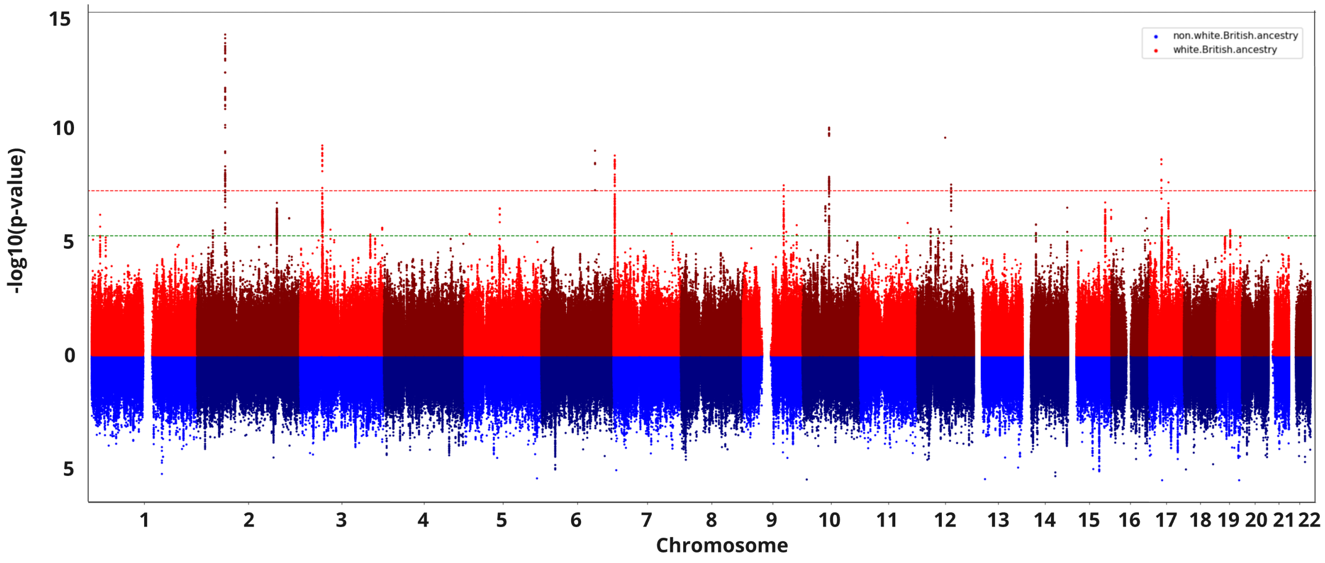

Figure S7: Miami plots showing significance of each variant's association with the right ACC region, for the annotation-free approaches. Results for subjects of white British ancestry are shown in red, while results for subjects of non-white British ancestry are shown in blue.

pheno	h2	se	lambdaGC	Mean Chi2	Intercept
dim1	0.0783	0.0174	1.0618	1.0752	1.0171
dim2	0.0767	0.0141	1.0496	1.0527	0.9961
dim3	0.0370	0.0150	1.0255	1.0282	1.0008
dim4	0.0208	0.0126	1.0345	1.0173	1.0016
dim5	0.0493	0.0152	1.0405	1.0445	1.0075
dim6	0.0233	0.0118	1.0165	1.0207	1.0036
dim7	0.0056	0.0158	1.0165	1.0204	1.0162
dim8	0.0213	0.0132	1.0165	1.0198	1.0041
dim9	0.0222	0.0128	1.0195	1.0134	0.9968
dim10	0.0163	0.0125	1.0135	1.0098	0.9976
dim11	0.0365	0.0142	1.0195	1.0223	0.9948
dim12	0.0236	0.0138	1.0105	1.0169	0.9996
dim13	0.0157	0.0119	1.0046	1.0035	0.9918
dim14	0.0189	0.0119	1.0225	1.0202	1.0063
dim15	0.0062	0.0115	1.0105	1.0123	1.0077
dim16	0.0085	0.0131	1.0255	1.0127	1.0065
dim17	0.0075	0.0136	1.0135	1.0115	1.0060
dim18	0.0308	0.0139	1.0195	1.0221	0.9991
dim19	0.0001	0.0141	1.0075	1.0081	1.0081
dim20	0.0587	0.0135	1.0466	1.0445	1.0014
dim21	0.0303	0.0122	1.0255	1.0178	0.9950
dim22	0.0031	0.0126	1.0046	1.0025	1.0002
dim23	0.0120	0.0129	0.9986	0.9981	0.9892
dim24	-0.0002	0.0160	1.0075	1.0133	1.0134
dim25	0.0367	0.0136	1.0075	1.0166	0.9893
dim26	0.0268	0.0142	1.0105	1.0095	0.9893
dim27	0.0051	0.0114	1.0075	1.0008	0.9970
dim28	0.0324	0.0138	1.0135	1.0136	0.9890
dim29	0.0213	0.0127	1.0165	1.0166	1.0008
dim30	-0.0159	0.0119	1.0105	1.0044	1.0163
dim31	0.0091	0.0114	1.0195	1.0106	1.0038
dim32	0.0028	0.0138	1.0135	1.0121	1.0101
dim33	-0.0212	0.0119	1.0075	1.0035	1.0193
dim34	-0.0205	0.0122	0.9957	0.9957	1.0110
dim35	0.0076	0.0126	0.9927	1.0005	0.9949
dim36	-0.0020	0.0120	0.9986	1.0000	1.0016
dim37	0.0223	0.0134	0.9986	1.0076	0.9910
dim38	0.0038	0.0129	1.0075	1.0024	0.9996
dim39	-0.0002	0.0124	1.0016	1.0056	1.0057

Table S1: Presents the heritability estimates computed using LDSC for each dimension of the latent space encoding the right ACC region. The reported metrics include SNP-based heritability (h^2), standard error (SE), genomic inflation factor (λ_{GC}), mean χ^2 statistic, and LDSC intercept.

Table S2: Genomic Loci Associated to the Right ACC Region[a]

Lead SNP	A1	Chr	BP	p_d	p_r	IndSigSNPs
rs2854108	A	1	19973920	2×10^{-10}	0.5	rs2854108;rs16822413
rs10168499	A	2	65987291	2×10^{-10}	0.3	rs10168499
rs7636227	A	3	52566682	5×10^{-9}	0.3	rs7636227
rs309588	A	5	82852578	6×10^{-11}	0.05	rs309588

[a] The tables show loci information, including the rsID of the lead SNP, allele 1 (A1), chromosome (Chr), base pair (BP) position of lead SNP on hc19, p-value for the discovery cohort (n=36,000) (p_d), for the replication cohort (n=5,395) (p_r) with the MOSTest, and independent significant SNPs (IndSigSNPs), from FUMA.

Table S3: Mapped Genes Associated to the Right ACC Region[b]

GENE	CHR	START	STOP	p
NT5DC2	3	52558386	52569070	3×10^{-7}
STAB1	3	52529354	52558511	6×10^{-7}
MINOS1-NBL1	1	19923473	19984549	2×10^{-6}

[a] This table lists the mapped genes locations regarding the p-values below the treshold 2×10^{-6}, for the discovery cohort (n=36,000), from MAGMA.

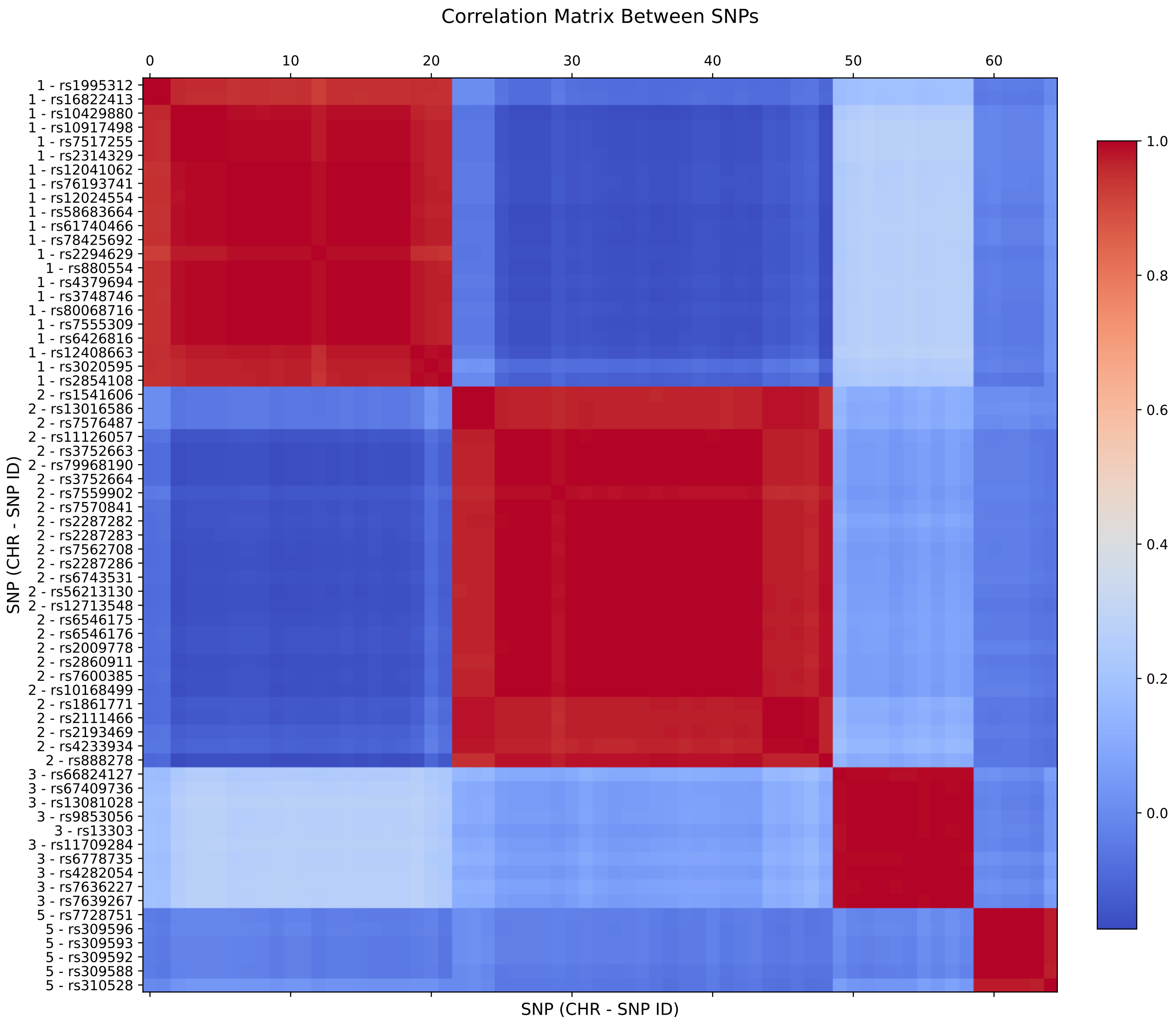

Figure S8: Correlation Matrix of Univariate GWAS z-Scores for Significant SNPs in the Right ACC region. The four loci identified by FUMA (see Table S2) are visible as four distinct red blocks. No strands were mislabeled, as evidenced by the absence of correlations near -1 within any of the LD blocks.

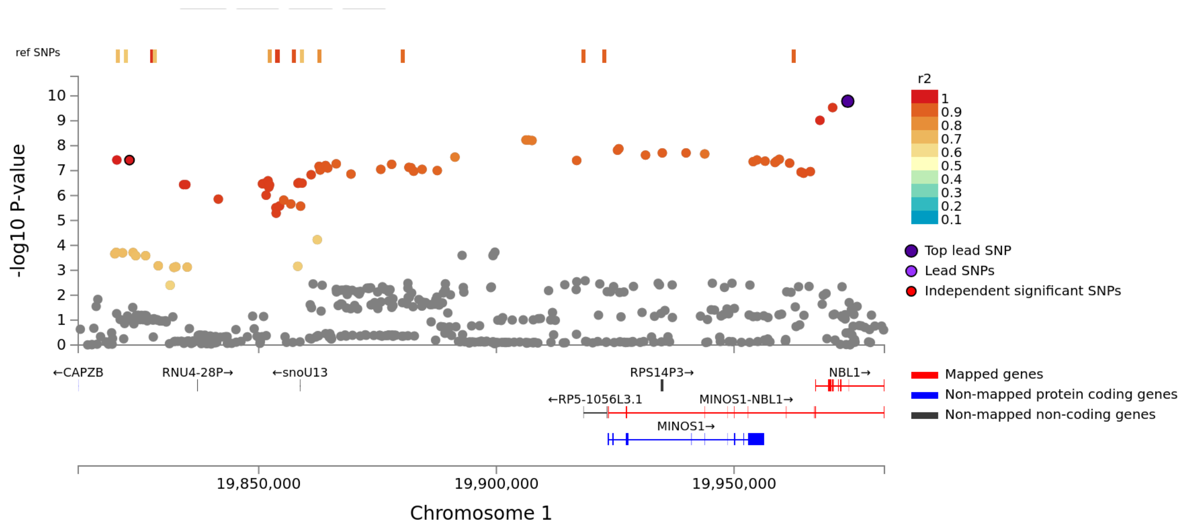

Figure S9: Regional plot obtained for the locus 1p36.13 (lead SNP rs2854108, $p_d = 2 \times 10^{-10}$), from FUMA.

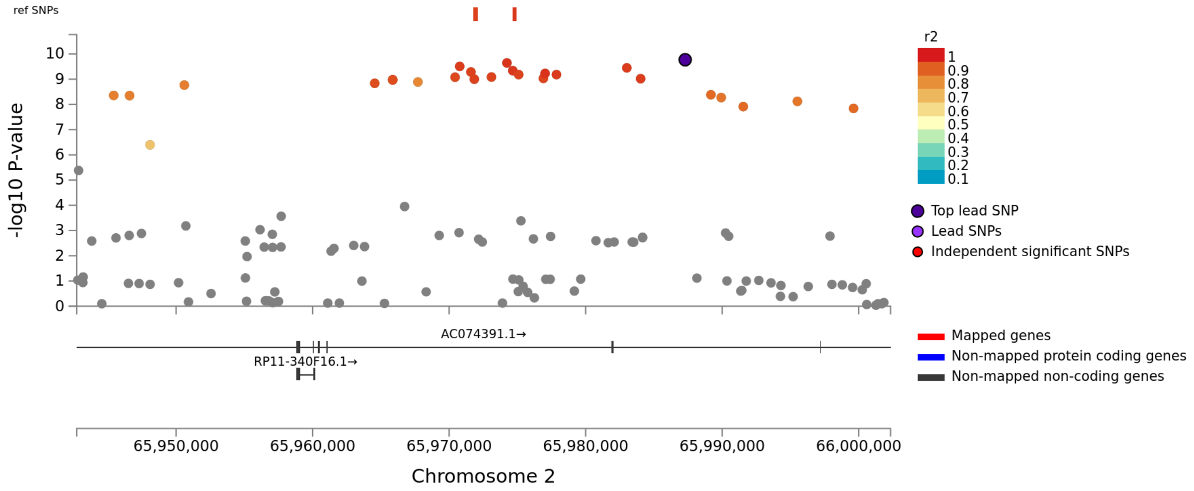

Figure S10: Regional plot obtained for the locus 2p14 (lead SNP rs10168499, $p_d = 2 \times 10^{-10}$), from FUMA.

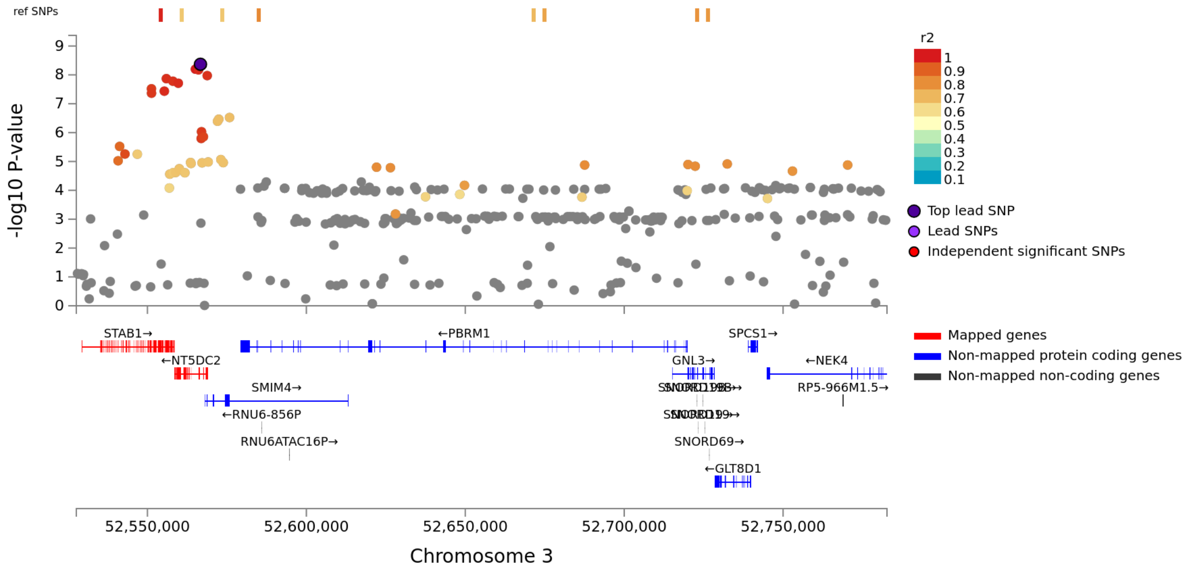

Figure S11: Regional plot obtained for the locus 3p21.1 (lead SNP rs7636227, $p_d = 5 \times 10^{-9}$), from FUMA.

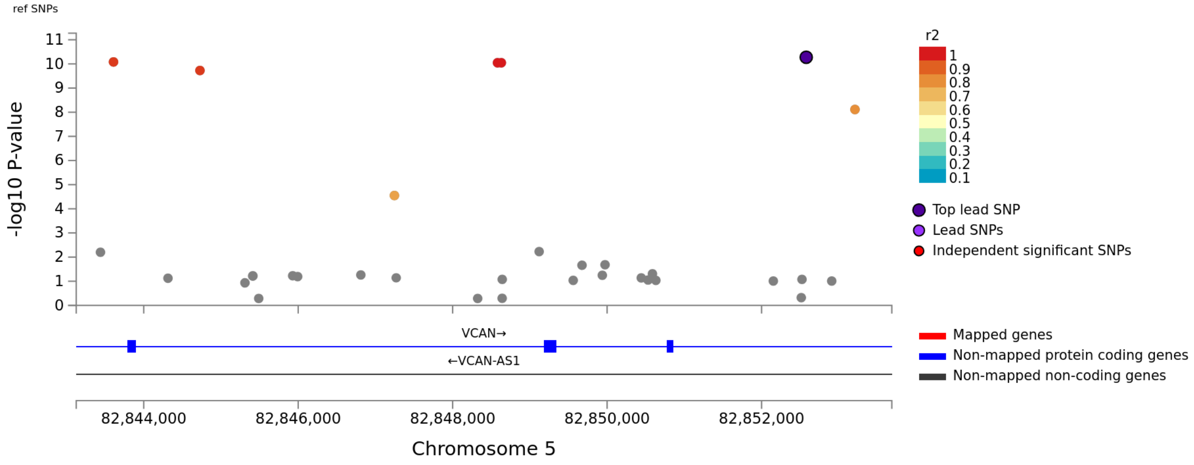

Figure S12: Regional plot obtained for the locus 5q14.3 (lead SNP rs309588, $p_d = 6 \times 10^{-11}$), from FUMA.

0.2 Left ACC Figures

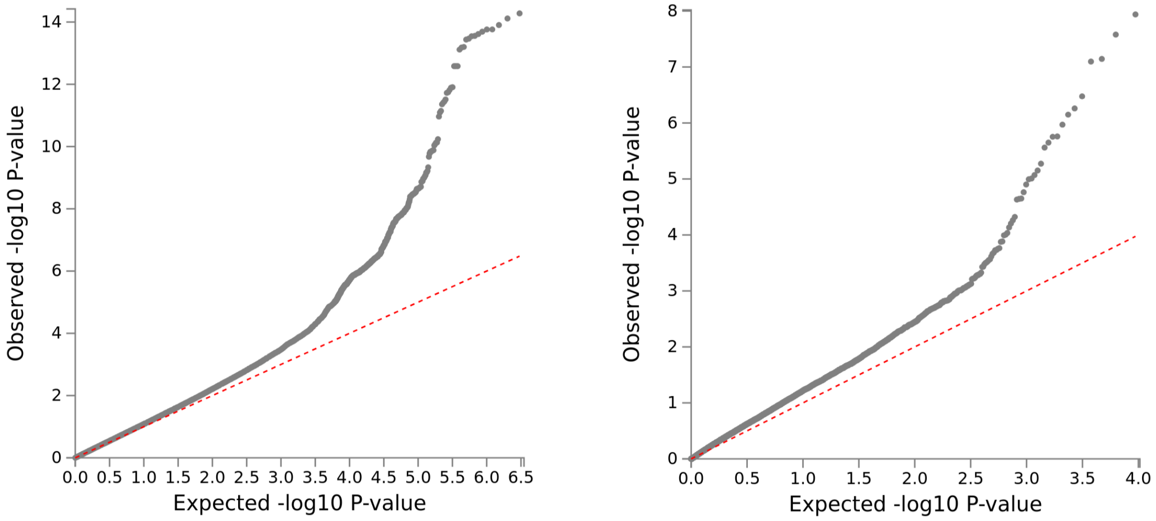

(a) SNP based QQ plot. (b) Gene based QQ plot.

Figure S13: QQ plots for the Left ACC region (from FUMA).

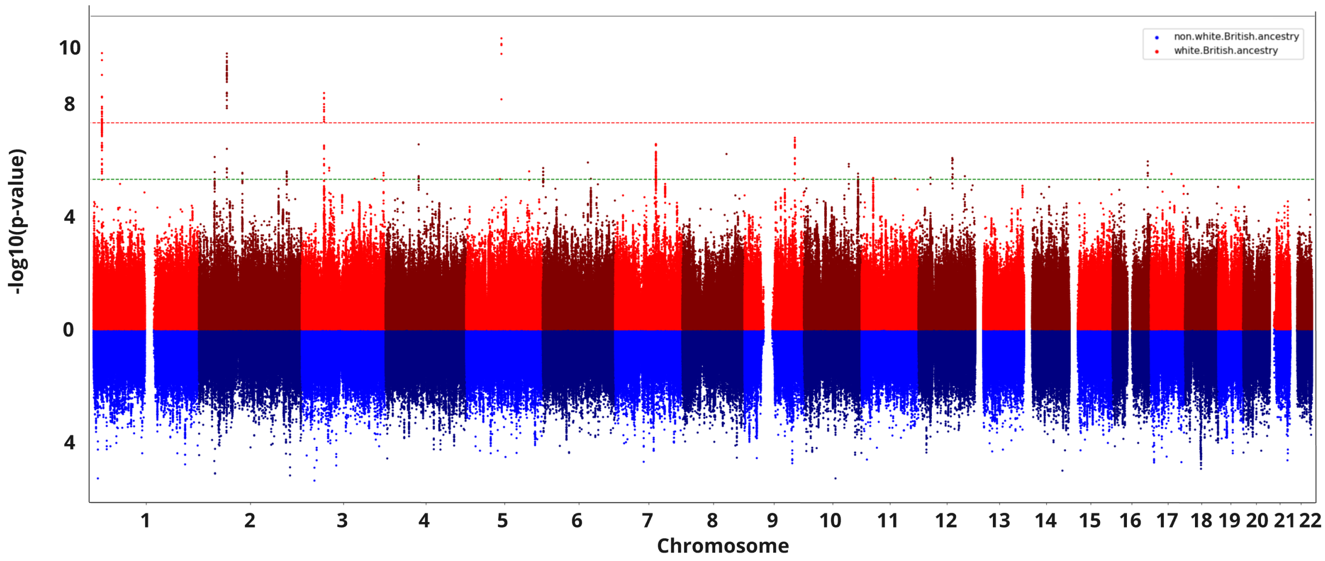

Figure S14: Miami plots showing significance of each variant's association with the left ACC region, for the annotation-free approaches. Results for subjects of white British ancestry are shown in red, while results for subjects of non-white British ancestry are shown in blue.

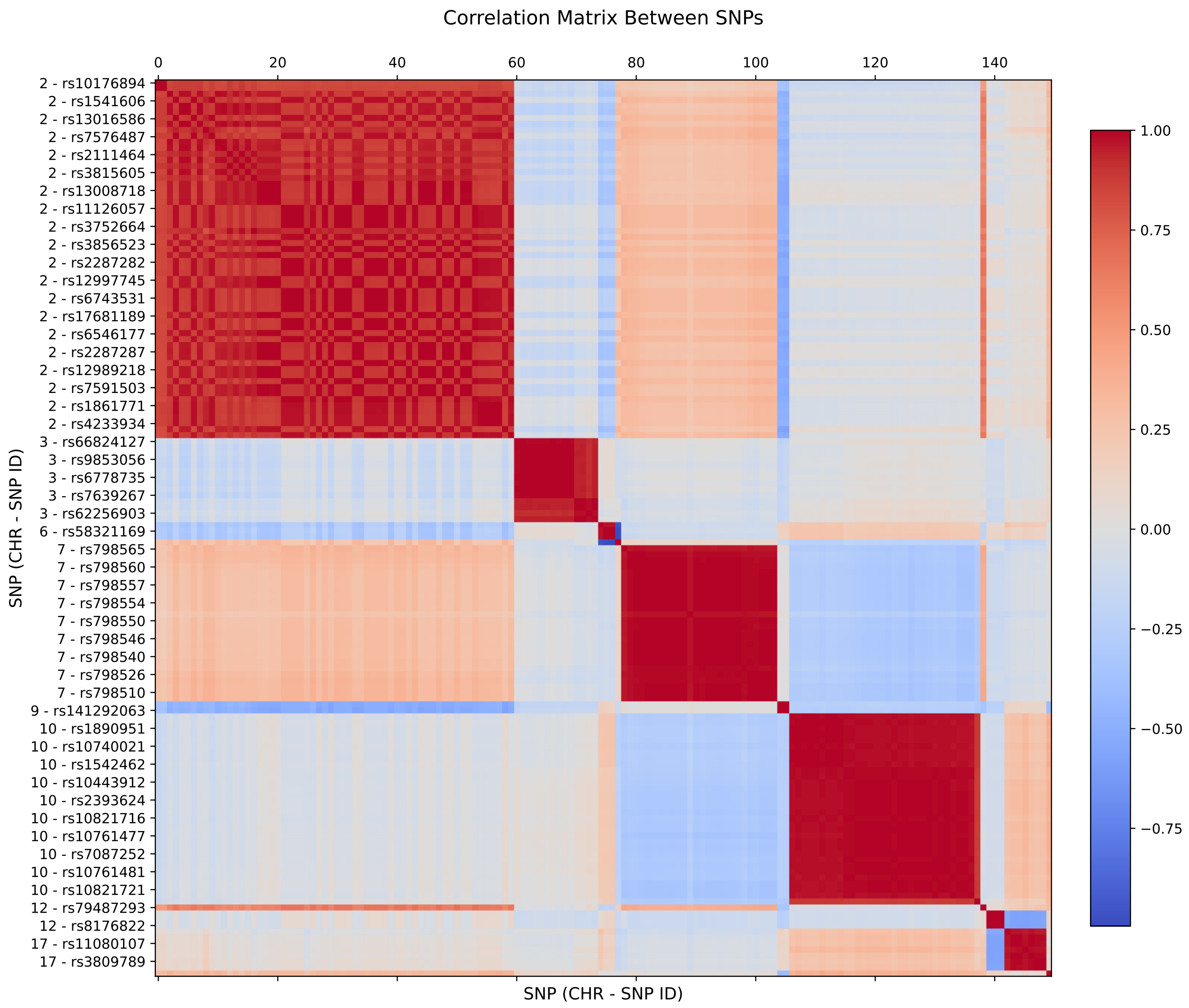

Figure S15: Correlation Matrix of Univariate GWAS Z-Scores for Significant SNPs in the Left ACC Region. The ten loci identified by FUMA (see Table **??**) are visible as ten distinct red blocks.

pheno	h2	se	lambdaGC	Mean Chi2	Intercept
dim1	0.1258	0.0207	1.0895	1.1079	1.0145
dim2	0.0376	0.0166	1.0315	1.0390	1.0111
dim3	0.0274	0.0136	1.0195	1.0296	1.0095
dim4	0.0151	0.0137	1.0165	1.0146	1.0033
dim5	0.0790	0.0184	1.0588	1.0586	0.9988
dim6	0.0695	0.0147	1.0496	1.0555	1.0041
dim7	0.0227	0.0139	1.0046	1.0121	0.9955
dim8	0.0153	0.0136	1.0165	1.0220	1.0106
dim9	0.0204	0.0124	1.0105	1.0144	0.9994
dim10	0.0114	0.0118	1.0105	1.0115	1.0032
dim11	0.0176	0.0150	1.0105	1.0086	0.9950
dim12	0.0595	0.0138	1.0405	1.0435	0.9996
dim13	0.0152	0.0127	1.0016	1.0127	1.0013
dim14	0.0146	0.0139	1.0135	1.0131	1.0021
dim15	0.0480	0.0152	1.0405	1.0482	1.0130
dim16	0.0142	0.0126	1.0135	1.0188	1.0085
dim17	0.0273	0.0170	1.0225	1.0237	1.0031
dim18	0.0348	0.0158	1.0105	1.0177	0.9914
dim19	0.0020	0.0126	1.0016	1.0027	1.0011
dim20	0.0543	0.0142	1.0375	1.0373	0.9967
dim21	0.0121	0.0135	1.0225	1.0156	1.0067
dim22	0.0288	0.0137	1.0105	1.0215	1.0001
dim23	0.0253	0.0114	1.0165	1.0164	0.9976
dim24	0.0248	0.0139	1.0225	1.0239	1.0052
dim25	0.0164	0.0135	1.0075	1.0191	1.0071
dim26	0.0074	0.0157	1.0075	1.0169	1.0115
dim27	0.0061	0.0130	1.0016	1.0051	1.0005
dim28	-0.0070	0.0122	1.0016	1.0038	1.0091
dim29	0.0137	0.0123	1.0165	1.0152	1.0052
dim30	0.0120	0.0148	0.9986	1.0057	0.9968
dim31	-0.0133	0.0124	0.9957	0.9972	1.0071
dim32	0.0090	0.0135	1.0016	0.9990	0.9925
dim33	0.0024	0.0124	0.9957	0.9968	0.9950
dim34	-0.0235	0.0113	0.9957	0.9976	1.0150
dim35	-0.0055	0.0136	1.0016	1.0021	1.0061
dim36	0.0455	0.0139	1.0255	1.0246	0.9911
dim37	0.0237	0.0134	1.0105	1.0155	0.9980
dim38	0.0238	0.0136	1.0165	1.0165	0.9983
dim39	0.0053	0.0126	0.9986	1.0029	0.9989
dim40	-0.0002	0.0128	0.9986	1.0003	1.0006
dim41	0.0101	0.0121	1.0135	1.0108	1.0034
dim42	0.0065	0.0143	0.9957	1.0013	0.9963

Table S4: Presents the heritability estimates computed using LDSC for each dimension of the latent space encoding the left ACC region. The reported metrics include SNP-based heritability (h^2), standard error (SE), genomic inflation factor (λ_{GC}), mean χ^2 statistic, and LDSC intercept.

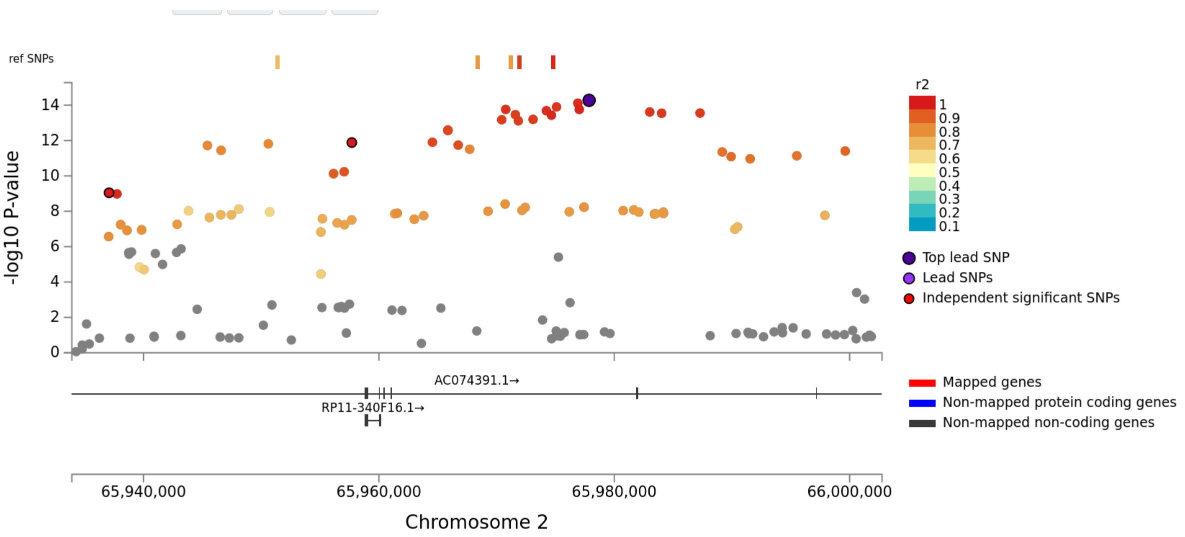

Figure S16: Regional plot obtained for the locus 2p14 (lead SNP rs2009778, $p_d = 6 \times 10^{-15}$), from FUMA.

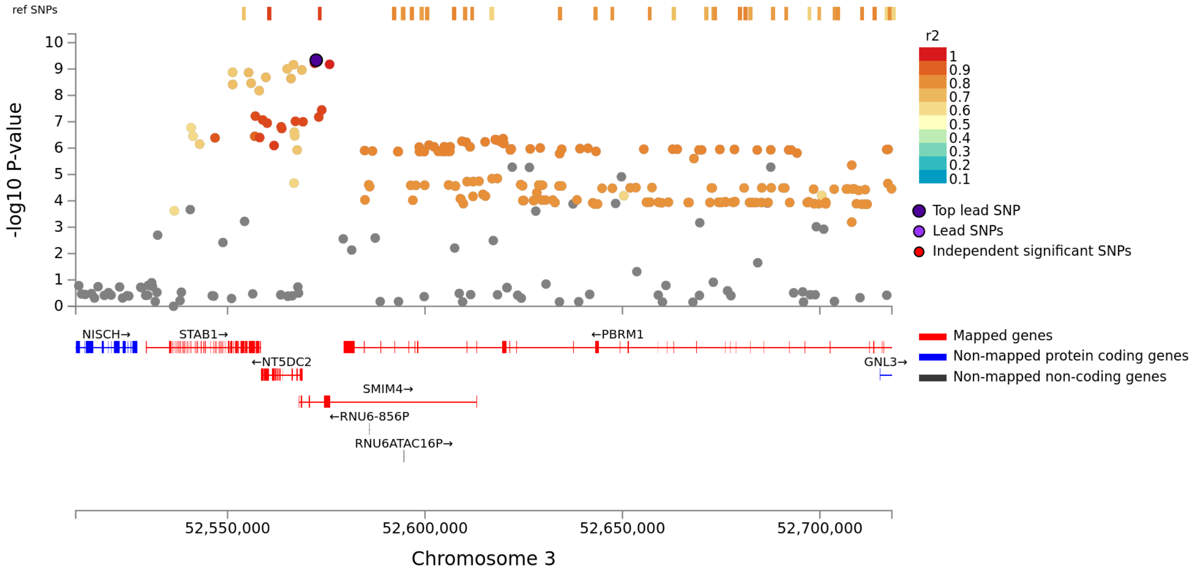

Figure S17: Regional plot obtained for the locus 3p21.1 (lead SNP rs6445528, $p_d = 5 \times 10^{-10}$), from FUMA.

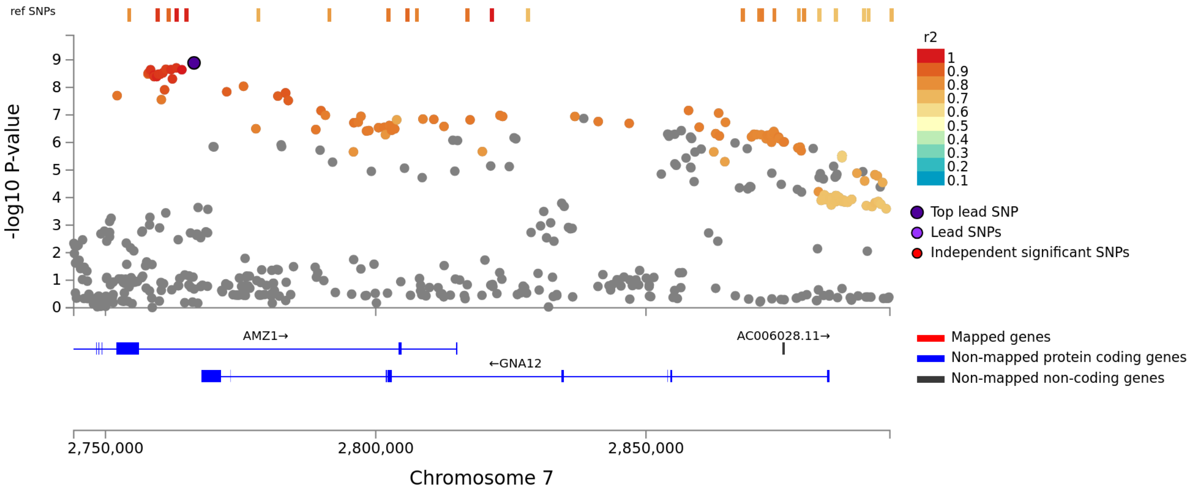

Figure S18: Regional plot obtained for the locus 7p22.3 (lead SNP rs798536, $p_d = 2 \times 10^{-9}$), from FUMA.

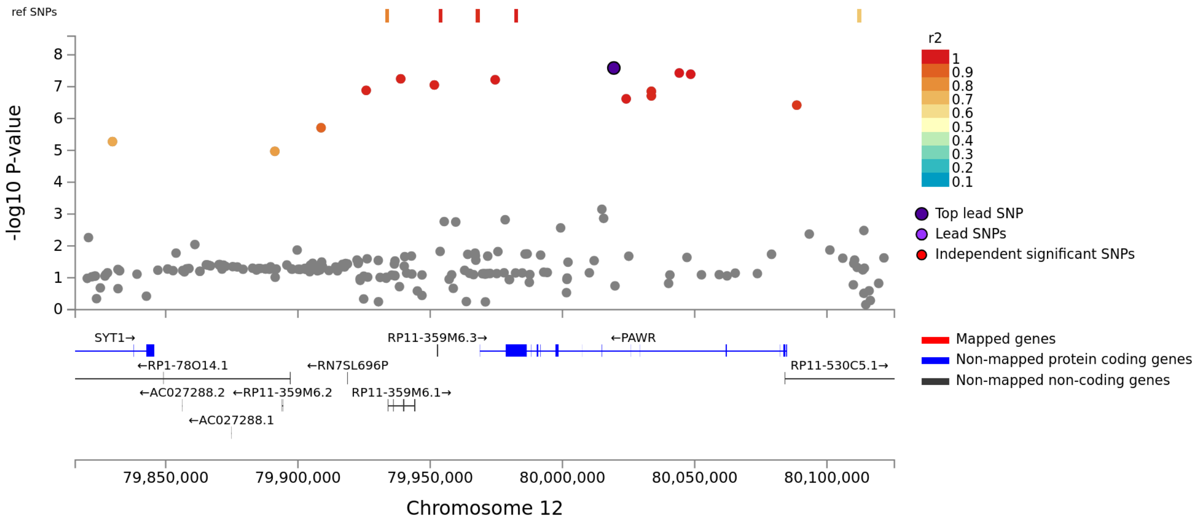

Figure S19: Regional plot obtained for the locus 12q21.2 (lead SNP rs4842267, $p_d = 3 \times 10^{-8}$), from FUMA.

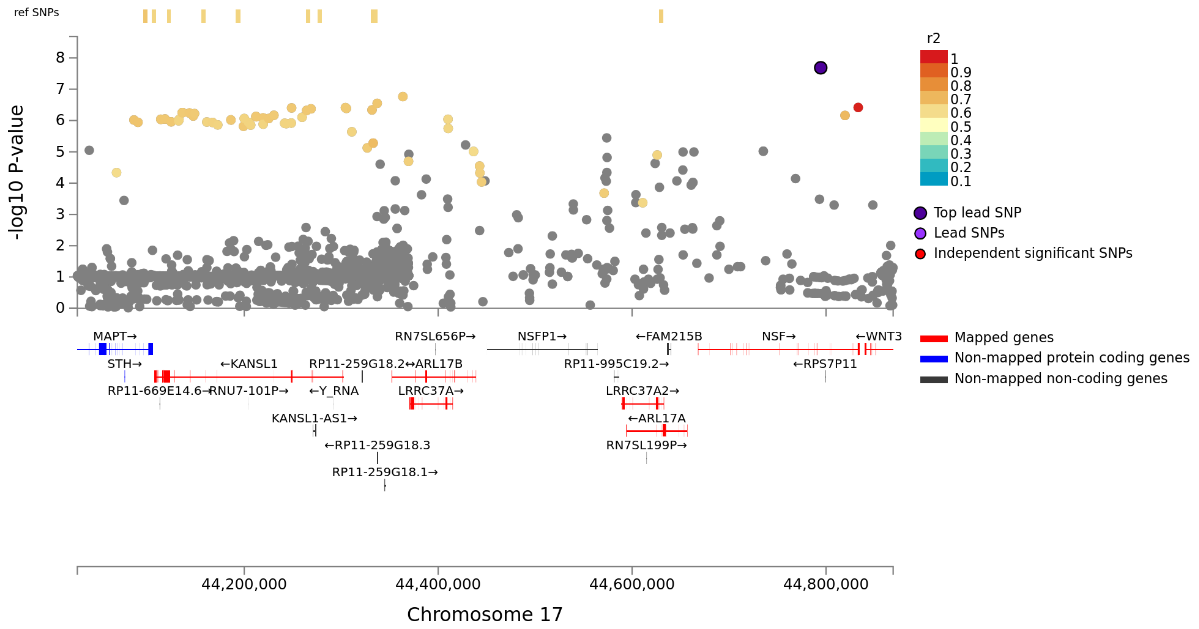

Figure S20: Regional plot obtained for the locus 17q21.31 (lead SNP rs12951057, $p_d = 2 \times 10^{-8}$), from FUMA.

Table S5: Gene Expression Across Developmental Stages[a]

Developmental Stage	β	β_{STD}	SE	p
Early prenatal	0.03	0.05	0.01	0.002
Early mid-prenatal	0.04	0.06	0.01	0.0002
Late mid-prenatal	0.04	0.06	0.01	0.002
Late prenatal	0.03	0.04	0.02	0.05
Early infancy	-0.1	-0.1	0.03	1
Late infancy	-0.1	-0.1	0.02	1
Early childhood	-0.02	-0.02	0.01	0.9
Late childhood	-0.1	-0.1	0.02	1
Adolescence	-0.04	-0.06	0.02	1
Young adulthood	-0.04	-0.05	0.02	1
Middle adulthood	-0.05	-0.07	0.02	1

[a] This table summarizes the gene expression effects (β), standardized effects (β_{STD}), and statistical significance p across various developmental stages, calulated on $N_{\text{GENES}} = 13,506$ genes from the MOSTest summary statistic with MAGMA, for the left ACC region.

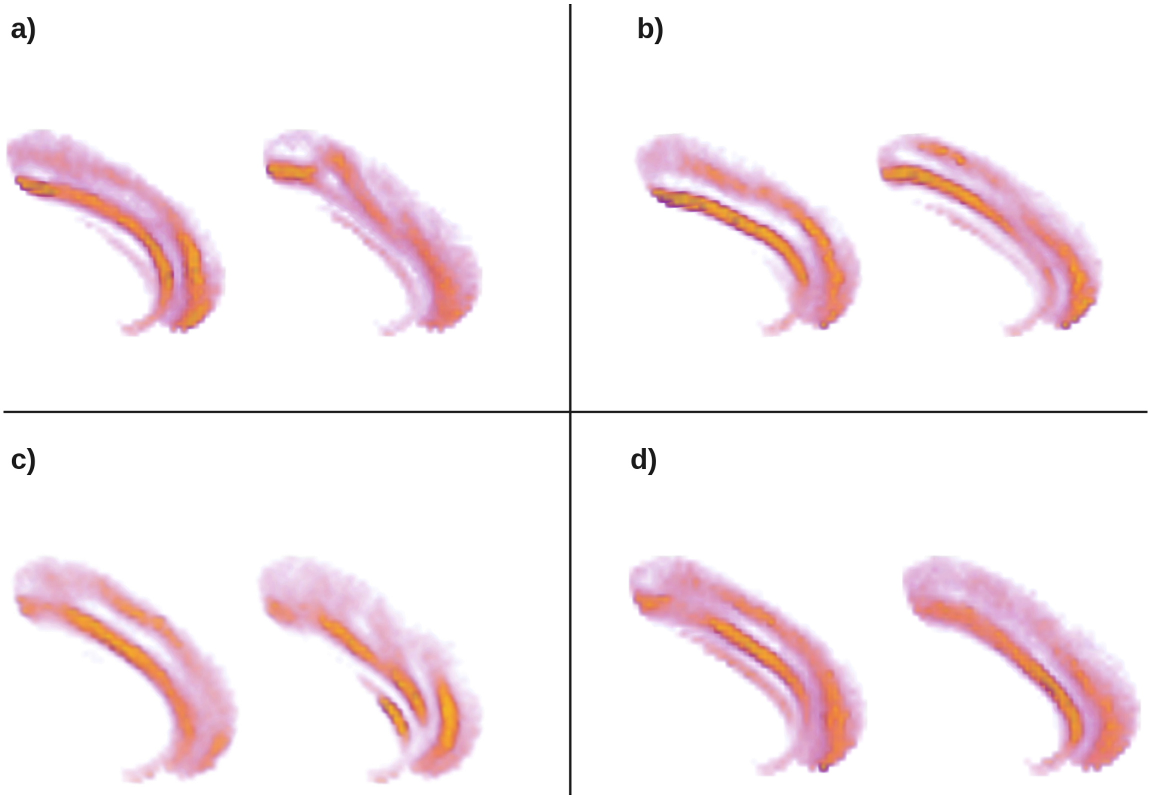

Figure S21: Illustration of cortical pattern variations predicted by regression on the latent space to estimate variant values. Extreme average patterns for the lead SNP **a)** rs2009778 ($p_d = 6 \times 10^{-15}$, $p_r = 0.004$, locus 2p14), **b)** rs798536 ($p_d = 2 \times 10^{-9}$, $p_r = 0.07$, locus 7p22.3), **c)** rs4842267 ($p_d = 3 \times 10^{-8}$, $p_r = 0.02$, locus 12q21.2), and **d)** rs12951057 ($p_d = 2 \times 10^{-8}$, $p_r = 0.09$, locus 17q21.31). Each subfigure highlights the structural differences associated with the respective genetic variants.

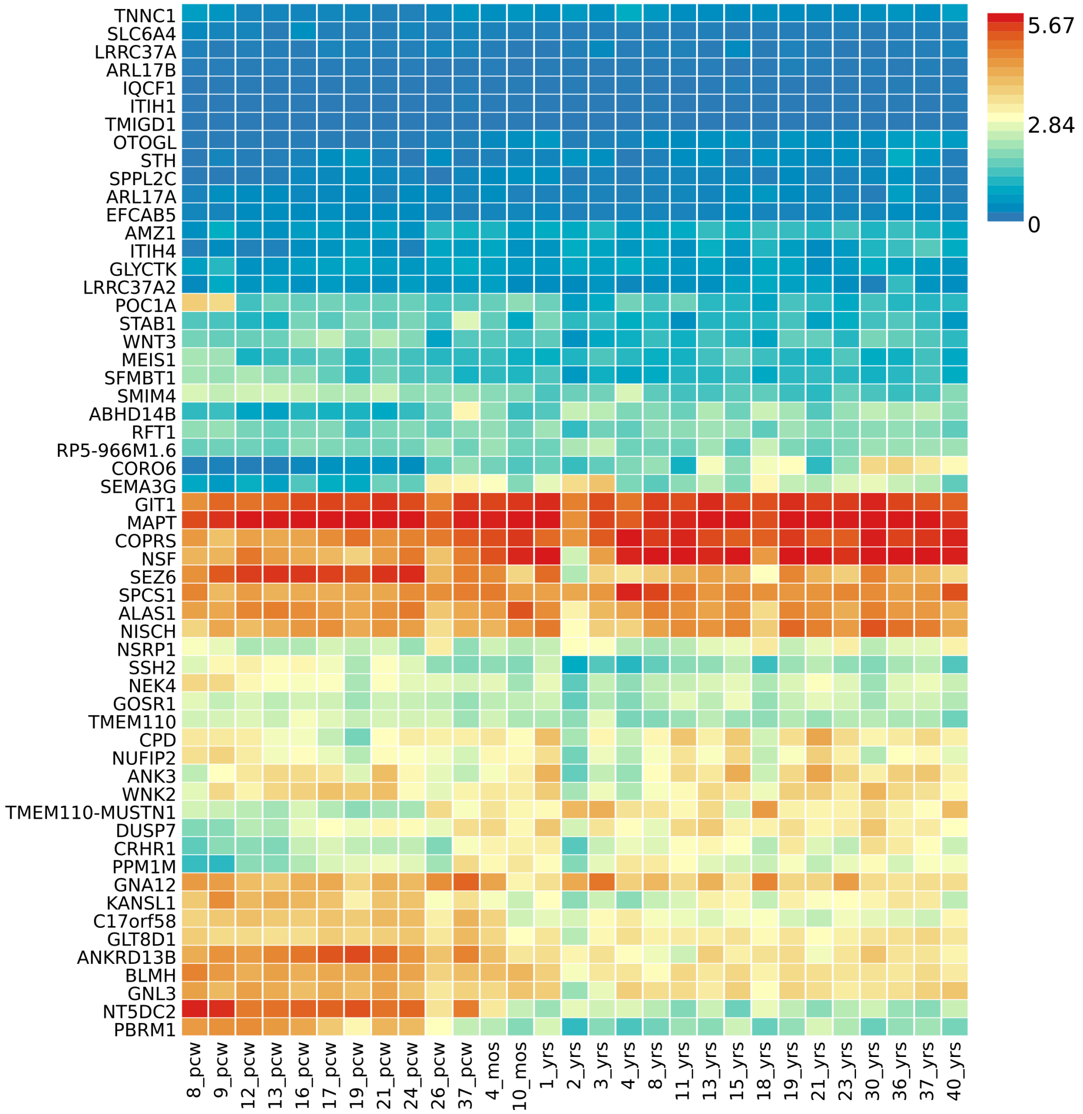

Figure S22: Average expression per label (log2 transformed) for the Left ACC region, clustered, from FUMA (BrainSpan 29 different ages of brain samples).

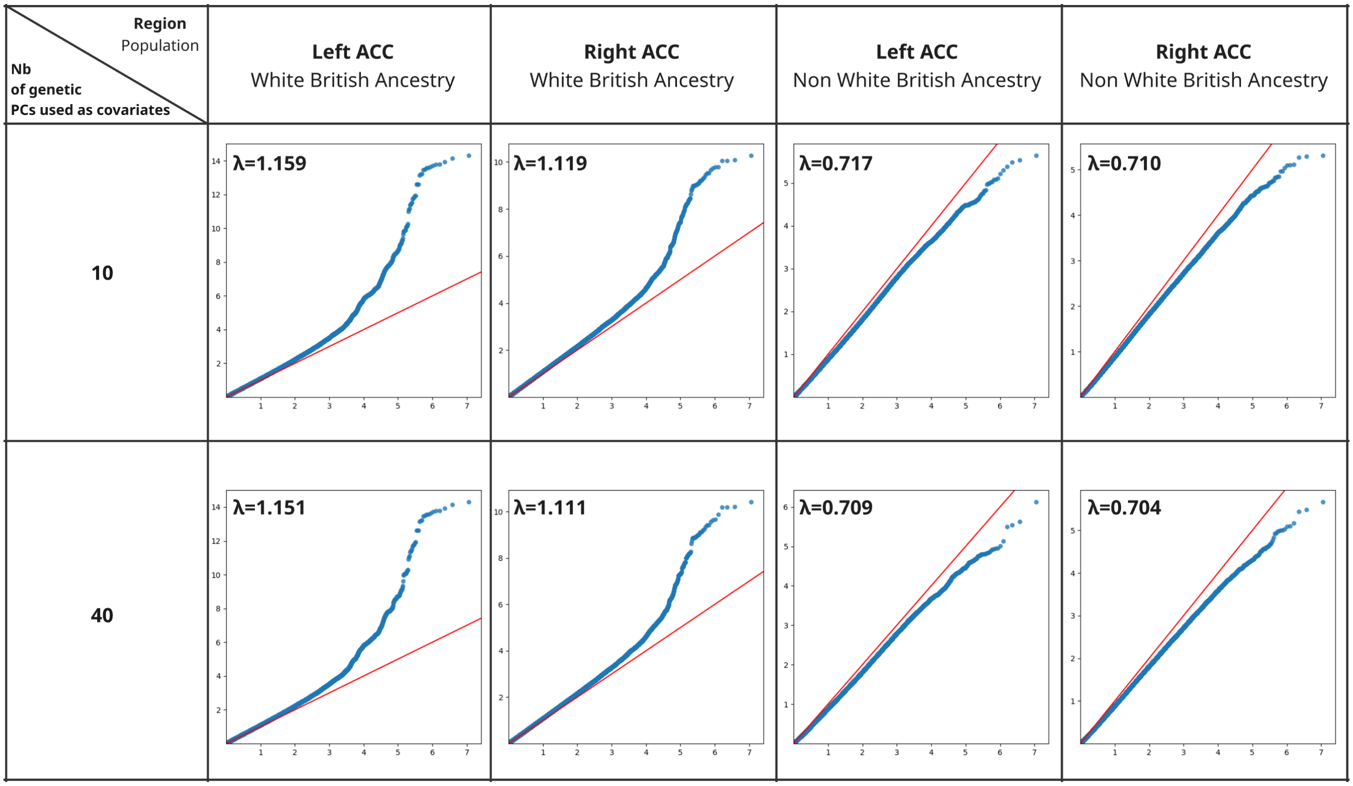

Figure S23: QQ plots and genomic inflation factors (λ) for SNP based tests with varying numbers of genetic PCs as covariates, for the left and right ACC, for both discovery and replication cohorts. The x-axis shows the expected $-\log_{10}(p)$ under the null, and the y-axis shows the observed $-\log_{10}(p)$.
